# Cross-talk between cancer and *Pseudomonas aeruginosa* mediates tumor suppression

**DOI:** 10.1038/s42003-022-04395-5

**Published:** 2023-01-06

**Authors:** Juliana K. Choi, Samer A. Naffouje, Masahide Goto, Jing Wang, Konstantin Christov, David J. Rademacher, Albert Green, Arlene A. Stecenko, Ananda M. Chakrabarty, Tapas K. Das Gupta, Tohru Yamada

**Affiliations:** 1 Department of Surgery, Division of Surgical Oncology, University of Illinois College of Medicine, Chicago, IL 60612 USA; 2 Department of Mathematics, Statistics and Computer Science, University of Illinois College of Liberal Arts and Sciences, Chicago, IL 60607 USA; 3 Department of Microbiology and Immunology and Core Imaging Facility, Loyola University Chicago, Maywood, IL 60153 USA; 4 Division of Pulmonary, Asthma, Cystic Fibrosis, and Sleep, Department of Pediatrics, Emory University School of Medicine, Atlanta, GA 30322 USA; 5 Department of Microbiology & Immunology, University of Illinois College of Medicine, Chicago, IL 60612 USA; 6 Richard & Loan Hill Department of Biomedical Engineering, University of Illinois College of Engineering, Chicago, IL 60607 USA; 7 Present Address: Department of Dermatology, University of Pennsylvania Perelman School of Medicine, Philadelphia, PA 19104 USA; 8 Present Address: General Surgery, Cleveland Clinic, Cleveland, OH 44195 USA

**Keywords:** Microbiology, Cancer

## Abstract

Microorganisms living at many sites in the human body compose a complex and dynamic community. Accumulating evidence suggests a significant role for microorganisms in cancer, and therapies that incorporate bacteria have been tried in various types of cancer. We previously demonstrated that cupredoxin azurin secreted by the opportunistic pathogen *Pseudomonas aeruginosa*, enters human cancer cells and induces apoptotic death^1–4^. However, the physiological interactions between *P. aeruginosa* and humans and their role in tumor homeostasis are largely unknown. Here, we show that *P. aeruginosa* upregulated azurin secretion in response to increasing numbers of and proximity to cancer cells. Conversely, cancer cells upregulated aldolase A secretion in response to increasing proximity to *P. aeruginosa*, which also correlated with enhanced *P. aeruginosa* adherence to cancer cells. Additionally, we show that cancer patients had detectable *P. aeruginosa* and azurin in their tumors and exhibited increased overall survival when they did, and that azurin administration reduced tumor growth in transgenic mice. Our results suggest host–bacterial symbiotic mutualism acting as a diverse adjunct to the host defense system via inter-kingdom communication mediated by the evolutionarily conserved proteins azurin and human aldolase A. This improved understanding of the symbiotic relationship of bacteria with humans indicates the potential contribution to tumor homeostasis.

## Abstract (web summary)

A bidirectional interaction between Pseudomonas aeruginosa (PA) bacteria and cancerous tumors results in an anti-tumor effect; cancer cells stimulate PA-secretion of azurin which promotes tumor cell production of aldolase A.

## Introduction

Microorganisms, particularly pathogenic bacteria, were used in the treatment of various types of human cancer over 100 years ago^5,6^ under the premise that a toxin produced by given pathogenic bacteria will inhibit the growth and spread of human cancer^7^. With the increasing knowledge of the role of the microbiota and the increasing incidence of cancer, treatment options using this approach have been reevaluated^8–13^.

We have been investigating the therapeutic role of microbes, such as the opportunistic pathogen *Pseudomonas aeruginosa*, in the management of human cancers^1–4,14,15^. Although *P. aeruginosa* is a major pathogen in cystic fibrosis (CF) causing significant morbidity and mortality^16^, CF patients have a lower incidence of melanoma and breast cancer than non-CF patients^17,18^. When supernatants of *P. aeruginosa* culture medium were co-incubated with tumor-derived J774A.1 macrophages, they induced apoptosis in a dose-dependent manner in J774A.1^2^. The supernatants of the culture medium contained a high concentration of the *P. aeruginosa*-secreted redox protein azurin^19,20^.

Azurin is a 14 kDa periplasmic copper protein containing 128 amino acids (aa) that are found in several types of bacteria and blue-green algae^21,22^. One of its known biological functions is as an electron transfer protein in anaerobic energy production via nitrogen fixation^23^. In vitro studies have demonstrated that azurin induces apoptotic cell death in a variety of cancer cells, with minimal or no effect on their normal counterparts^1–3,14,24–26^. Upon preferential entry into cancer cells, azurin binds to the tumor suppressor protein p53 and induces caspase-mediated apoptosis^1,3,27^. Human xenotransplant studies in athymic mice showed that systemic administration of azurin inhibited tumor growth without significant adverse effects on the host^1,3,28^. In addition, we previously identified the active peptide fragment of azurin (i.e., p28 or Azu28)^24,29^, and the efficacy of this 28-aa cell-penetrating peptide p28 has been extensively investigated both experimentally and clinically^30–32^.

Although the clinical application and usefulness of azurin and its 28-aa peptide have been recognized, the physiological roles of *P. aeruginosa* in relationship with the host when it encounters cancer cells—which in turn inhibit cancer growth and spread—have not been investigated. The primary objective of this study is to demonstrate the interaction of *P. aeruginosa* with human cancer cells and its role in tumor homeostasis. Besides highlighting the importance of this specific bacterial-cancer interaction, we show for the first time the bidirectional regulation of bacterial-cancer communication in relation to the potential for *P. aeruginosa*-secreted azurin to inhibit tumor growth. Our observations suggest that these interactions may act as diverse adjuncts to the host defense system via inter-kingdom communication.

## Results

### High levels of *P. aeruginosa* azurin in the sera of CF patients

Based on the frequent colonization of CF patients with *P. aeruginosa*, the lower incidence of melanoma and breast cancer in CF than non-CF patients^17,18^ and previous descriptions of cancer-specific effects of azurin^1,28,33–36^, we hypothesized that azurin from *P. aeruginosa* plays a role in tumor development. To test our hypothesis that a bacterial protein, azurin, is detectable in human sera and that there is a significant elevation of azurin levels in CF patients, we first compared the serum levels of azurin in CF patients with chronic *Pseudomonas* infection to those in healthy volunteers with no disease (controls; see Supplementary Fig. 1a for demographic information) and found that azurin levels in CF patients were significantly higher than those in controls (Supplementary Fig. 1b). The serum level of azurin was correlated with the age of CF patients (Supplementary Fig. 1c). This clinical evidence showed that CF patients have significantly elevated levels of circulating azurin.

### *P. aeruginosa* azurin secretion is stimulated by cancer cells

**Fig. 1:**
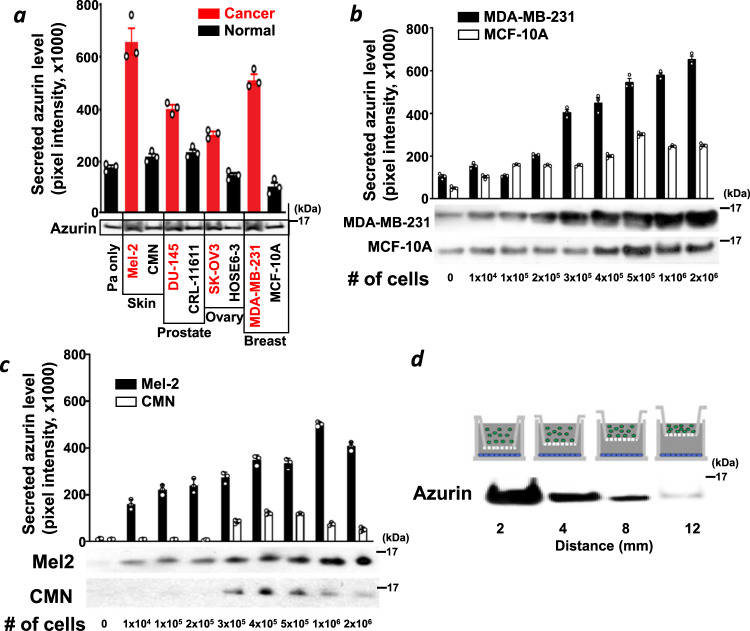
Azurin secretion is stimulated by human cancer cells. **a** Induction of azurin secretion by *P. aeruginosa* in the presence of host cells. Human cells and *P. aeruginosa* were co-incubated for 30 min at a concentration of 500,000 human cells/ml and *P. aeruginosa* (Pa) at an optical density (OD) = 0.3. Human cell lines derived from various tissues and tumors were used: melanoma Mel-2, congenital melanocytic nevus CMN, prostate cancer DU-145, normal prostate CRL-11611, ovarian cancer SK-OV3, normal ovary HOSE6-3, breast cancer MDA-MB-231, and normal breast MCF-10A. Secretion of azurin by *P. aeruginosa* into the culture supernatant was assessed by western blot analysis; the graph shows the observed band intensities determined by a densitometer UN-SCAN-IT gel version 5.1. The mean+SE values were calculated for skin, prostate, ovarian, and breast cell pairs. **b**, **c** Azurin secretion is cancer cell dose-dependent. *P. aeruginosa* secretes higher levels of azurin in the presence of human breast cancer MDA-MB-231 (**b**) and melanoma Mel-2 cells (**c**) than in the presence of melanocytes (CMN) or non-malignant MCF-10A breast cells, in a dose-dependent manner. Cancer and normal/non-malignant cells were co-incubated with *P. aeruginosa* for 30 min at concentrations ranging from 0 to 2,000,000 cells/ml and *P. aeruginosa* at OD = 0.3. Secretion of azurin by *P. aeruginosa* into the culture supernatant was assessed by western blot analysis; the graph shows the observed band intensities. The mean+SE values were calculated. **d** Soluble extracellular factors stimulate azurin secretion in a distance-dependent manner. Mel-2 and *P. aeruginosa* cells were separated by a permeable Transwell^®^ insert membrane with a pore size of 0.4 μm. In this co-culture system, *P. aeruginosa* (OD = 0.3) and Mel-2 cells (3,000,000 cells/ml) were separated at distances ranging from 2 mm to 12 mm and incubated for 30 min at 37 °C (inset figure: schematic of the co-culture system. Green: *P. aeruginosa*, blue: Mel-2). Secretion of azurin by *P. aeruginosa* into the culture supernatant was assessed by western blot analysis; the graph shows the observed band intensities.

To investigate how host cells, either human cancer cells or their corresponding normal counterparts, affect *P. aeruginosa* azurin secretion, azurin secretion levels were measured in *P. aeruginosa*-host cell cultures. Azurin secretion was significantly higher, as were the transcription levels of the azurin-encoding gene *azu*, in the presence of various human cancer cell lines than in the presence of their normal counterparts (Fig. 1a, Supplementary Fig. 2). The evidence that the incidence rates of melanoma and breast cancer are lower in CF patients^17,18^ and that these two types of cancers showed high contrast in azurin secretion in our experimental systems^1,3^ led us to focus on these cancers for the remainder of this study. Azurin secretion by *P. aeruginosa* was positively correlated with the number of human breast cancer (Fig. 1b) or melanoma (Fig. 1c) cells, and azurin secretion was significantly lower when *P. aeruginosa* was incubated with normal breast cells or benign nevus (congenital melanocytic nevus, CMN) cells (Fig. 1a–c). These results suggest that *P. aeruginosa* preferentially secretes azurin in the presence of cancer cells and that this effect has a dose-dependent relationship with the population of cancer cells.

We next determined whether the bacteria-cancer interaction-mediated modulation of azurin secretion requires direct cell-to-cell contact between *P. aeruginosa* and cancer cells. Secreted azurin levels were highest at the shortest distance (2 mm) between the bacteria and cancer cells and quite low at distances greater than 12 mm, showing an inversely proportional correlation (Fig. 1d). Despite the lack of physical contact, azurin secretion was elicited, thereby indicating that a soluble factor secreted by Mel-2 cells may act as a stimulus. Changes in azurin levels were not due to *P. aeruginosa* contamination from top to bottom wells (Supplementary Fig. 3). These results suggest the existence of at least one soluble agent originating from cancer cells that exerts a concentration-dependent stimulatory effect on azurin secretion.

### Cancer cells secrete aldolase A in response to azurin

The significant stimulation of azurin secretion by malignant host cells compared to that by their normal counterparts suggested that these cell groups differed in the secretion of a host factor. To identify any proteins that were differentially secreted in the presence of *P. aeruginosa*, we used a mass spectrometry-based proteomic approach. We found that human aldolase A was released extracellularly into the culture medium when human cancer cells or their normal counterparts were co-incubated with *P. aeruginosa* (Supplementary Fig. 4a–c).

**Fig. 2:**
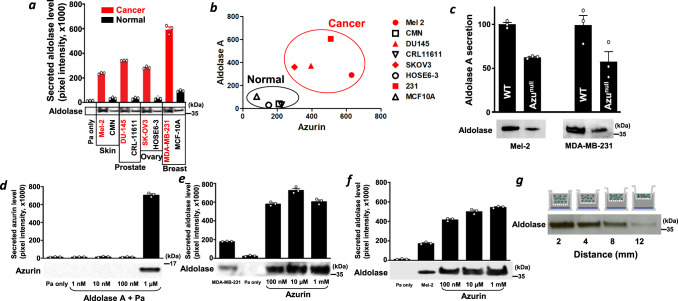
A bidirectional interaction between P. aeruginosa and human cancer cells. **a** Induction of aldolase A secretion by host cells in the presence of *P. aeruginosa*. Human host cells (cancer and normal) and *P. aeruginosa* were co-incubated for 30 min at a concentration of 500,000 human cells/ml with *P. aeruginosa* at OD = 0.3. Aldolase secretion varied between the co-cultures with cancer and normal cells. Secretion of azurin and aldolase into the culture supernatant was assessed by western blot analysis; the graph shows the observed band intensities. **b** Correlation between the aldolase A and azurin levels in co-cultures of *P. aeruginosa* with cancer or normal cells. **c** Cancer cells (Mel-2 and MDA-MB-231) and *P. aeruginosa* (WT and azu^null^ mutant) were co-incubated for 30 min at a concentration of 500,000 human cells/ml with *P. aeruginosa* at OD = 0.3. Secretion of aldolase into the culture supernatant was assessed by western blot analysis. **d** *P. aeruginosa* (OD = 0.3) was treated with purified aldolase A protein at concentrations of 1 nM, 10 nM, 100 nM, and 1 μM for 30 min. Treatment with 1 μM aldolase A stimulated azurin secretion from *P. aeruginosa*, suggesting that aldolase A is a stimulatory factor for azurin secretion. Secretion of azurin by *P. aeruginosa* into the culture supernatant was assessed by western blot analysis; the graph shows the observed band intensities. MDA-MB-231 (**e**) and Mel-2 (**f**) cells were treated with purified azurin protein at concentrations of 100 nM, 10 μM, and 1 mM for 30 min. Secretion of aldolase A by cancer cells into the culture supernatant was assessed by western blot analysis; the graph shows the observed band intensities. *P. aeruginosa* (Pa only) did not show any signal as it did not secret any proteins that cross-react with anti-aldolase A antibody. **g** Aldolase A secretion in the presence of *P. aeruginosa* is distance-dependent. Mel-2 cells and *P. aeruginosa* were separated with a permeable Transwell® insert. In this co-culture system, *P. aeruginosa* (OD = 0.3) and Mel-2 cells (3,000,000 cells/ml) were separated at distances ranging from 0 mm to 12 mm and incubated for 30 min at 37 °C. Aldolase A secretion decreased as the distance between the two cell populations increased. Secretion of aldolase by Mel-2 cells into the culture supernatant was assessed by western blot analysis; the graph shows the observed band intensities.

Aldolase, also called fructose-bisphosphate aldolase (FBA), is a glycolytic enzyme involved in the Embden-Meyerhof-Parnas glycolytic pathway and gluconeogenesis and is highly conserved in bacterial, archaeal, and eukaryotic organisms. Human aldolase A secretion by human cancer cells in the presence of *P. aeruginosa* was significantly higher than that by their normal counterparts (Fig. 2a). Changes in aldolase A secretion were not due to alteration of the cell growth rate or induction of toxicity in cancer cells (Supplementary Fig. 5a, b). The secretion levels of aldolase A in culture medium were related to the secretion levels of azurin when human malignant and benign cells derived from various tumors and tissues were co-incubated with *P. aeruginosa* (Fig. 2b). To address the role of azurin on aldolase A secretion, three experimental approaches were taken: aldolase A secretion induced by (1) wild type (WT) and *azu* gene deleted mutant *P. aeruginosa*^37^, (2) the non-pathogenic *E. coli* expressing azurin gene, and (3) recombinant azurin protein. First, aldolase A secretion was measured when cancer cells were co-incubated with WT and azu^null^ mutant *P. aeruginosa*. Aldolase A secretion induced by WT *P. aeruginosa* was significantly higher than azu^null^ mutant, but azu^null^ mutant can still induced aldolase A secretion from cancer cells (Fig. 2c, Supplementary Fig. 6). Next, the interaction of the non-pathogenic *E. coli* laboratory strain JM109 (a K-12 derivative) transformed with the azurin-encoding gene with cancer cells was evaluated to determine whether the secretion of azurin and aldolase A is specific to the *P. aeruginosa* strain we used. *E. coli* expressing *azu* gene secreted a considerable amount of azurin in the presence of Mel-2 cells. Additionally, aldolase A secretion from Mel-2 cells was induced by *azu*-expressing *E. coli* (Supplementary Fig. 7), suggesting that (1) azurin is a major inducer of aldolase A secretion, but not a sole inducer from *P. aeruginosa*, and (2) *E. coli* has a secretion mechanism similar to that of *P. aeruginosa*.

To further investigate the impact of human aldolase A on azurin secretion during the interaction of bacteria and human cancer cells, *P. aeruginosa* cells were cultured in the presence of purified human aldolase A (purity >95%), and azurin secretion was measured by western blotting. Exposure of *P. aeruginosa* to aldolase A induced azurin secretion (Fig. 2d). Conversely, when breast cancer and melanoma cells were cultured in the presence of purified azurin, these cancer cells secreted a considerable amount of human aldolase A into the culture medium (Fig. 2e, f) without increasing gene expression or intracellular levels of aldolase A, suggesting that aldolase A secretion induced by azurin occurs in a transcription-independent manner (Supplementary Fig. 8a, b). Similar to the pattern of azurin secretion shown in Fig. 1d, the levels of secreted aldolase A were highest at the shortest distance (2 mm) between *P. aeruginosa* and cancer cells (Fig. 2g), that secreted azurin has a major role to induce aldolase A secretion, and their interaction is bidirectional and concentration density-dependent inter-communication between *P. aeruginosa* and cancer cells via azurin and aldolase A (Fig. 2).

### Aldolase A promotes *P. aeruginosa* localization on cancer cells

**Fig. 3:**
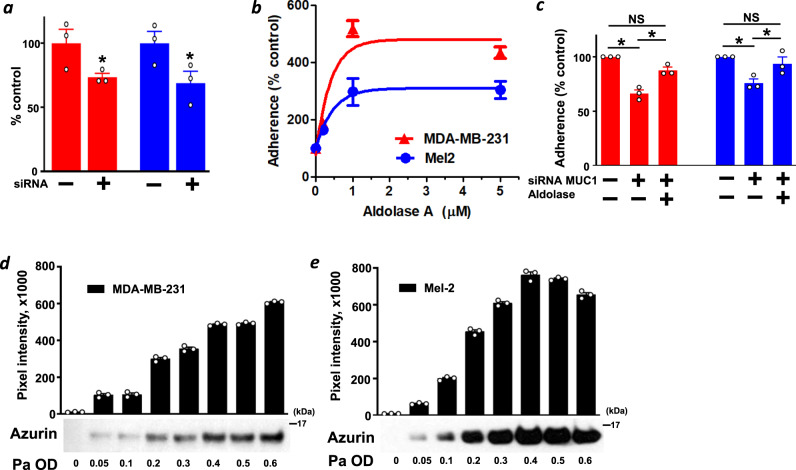
Effect of aldolase A on *P. aeruginosa* cell adhesion. **a** Adherence assays were performed essentially as previously described^38^. Monolayer MDA-MB-231 (red) and Mel-2 (blue) cells were compared to siRNA-induced silencing of aldolase gene in the cancer cell lines (+) when they were co-incubated with *P. aeruginosa* for 2 h. To assess total cell association, monolayers were washed to remove unbound *P. aeruginosa* and were then disrupted and homogenized in 0.1% saponin/PBS. *P. aeruginosa* cells were counted by serial dilution of the homogenized suspensions and subsequent determination of colony-forming units (CFU) by plating on LB agar. Control (–) expressed as 100%. Mean+SE, *P < 0.05. **b** Monolayer MDA-MB-231 (red) and Mel-2 (blue) cells were co-incubated with *P. aeruginosa* in the presence or absence of exogenous aldolase A for 2 h. Similar to above, total *P. aeruginosa* association on cancer cells were counted by plating on LB agar. Control (0 µM aldolase A) expressed as 100%. **c** Monolayer MDA-MB-231 (red) and Mel-2 (blue) cells were compared to siRNA-induced silencing of MUC1 genes in the cancer cell lines (+) when they were co-incubated with *P. aeruginosa* in the presence or absence of exogenous aldolase A at 200 nM for 2 h. Total *P. aeruginosa* association on cancer cells were counted by plating on LB agar. *P < 0.05, NS: not significant. Azurin secretion is *P. aeruginosa* dose-dependent. MDA-MB-231 (**d**) and Mel-2 (**e**) cells (500,000 cells/ml) were co-incubated with *P. aeruginosa* at concentrations corresponding to an OD ranging from 0.0 to 0.6. Secretion of azurin by *P. aeruginosa* into the culture supernatant was assessed by western blot analysis; the graph shows the observed band intensities. The mean+SE values were calculated.

Although the cytosolic role of aldolase in glycolysis and gluconeogenesis has long been recognized, aldolase is reportedly also involved in host cell adhesion and biofilm formation in bacteria and parasites such as *Streptococcus*, *Neisseria*, *Toxoplasma* and *Plasmodium*^38–42^. This prompted us to investigate whether aldolase A secreted by human cancer cells plays a similar biological role in the adherence of *P. aeruginosa* to cancer cells^38,43^. First, we tested whether silencing aldolase A gene will alter the adherence of *P. aeruginosa* to cancer cells. The *P. aeruginosa* adhesion assay showed that siRNA-induced silencing aldolase A in MDA-MB-231 and Mel-2 cells (Supplementary Fig. 9a) significantly reduced the adherence of *P. aeruginosa* (Fig. 3a). Conversely, in the presence of purified human aldolase A, *P. aeruginosa* exhibited significantly increased adherence to cancer cells, and the increase was dose-dependent and saturable (>1 µM) (Fig. 3b). It has been reported that MUC1, an O-glycosylated membrane-tethered mucin on cancer cells, interacts with *P. aeruginosa* through flagellin^44,45^. Muc1^‒/‒^ animals displayed ~50% less adherence of *P. aeruginosa* in the lungs compared with Muc1^+/+^ mice^46^. Hence, we tested the effect of siRNA-induced MUC1 silencing in cancer cells (Supplementary Fig. 9b) by our *P. aeruginosa* adhesion assay. Silencing MUC1 in MDA-MB-231 and Mel-2 cells significantly reduced the adherence of *P. aeruginosa* (Fig. 3c). Moreover, when recombinant aldolase A was added to MUC1 silenced cancer cells, the adhesion rate of *P. aeruginosa* was similar to control values, suggesting that aldolase-mediated adherence of *P. aeruginosa* was, at least partly, independent of MUC1-mediated adhesion.

These results suggest that aldolase A induces *P. aeruginosa* adhesion and colonization in cancer cells. Although mucins have been suggested as the preferred sites for adherence and colonization of *P. aeruginosa* on host cells^47^, *P. aeruginosa* can also bind to several other host proteins. These proteins include aldolase, and this binding is mediated by hydrophobic interactions^48^.

### Azurin secretion is *P. aeruginosa* density dependent

The complexity of *P. aeruginosa* genome reflects evolutionary adaptation permitting it to thrive in diverse environments, including eukaryotic hosts, with which it has coexisted for millions of years^49^. *Pseudomonas spp*. are renowned among prokaryotes for their complex quorum sensing (QS) systems that regulate biofilm formation^50,51^. In general, bacterial QS of stimuli and responses correlates to population density^52^. Based on our data, *P. aeruginosa* secretes low levels of azurin due to the presence of only a small population of either cancer cells (Fig. 1b, c) or *P*. *aeruginosa* (Fig. 3d, e). This finding suggests that a high concentration of aldolase A due to a large population of cancer cells enhances azurin secretion by increasing bacterial adherence and the density of the bacterial population. However, this mode of density sensing in *P. aeruginosa* is likely independent of the intercellular QS communication system since it has been previously shown that azurin is actively expressed in *Pseudomonas* mutants carrying mutations in the Gac/Rsm system that activates the QS machinery primarily by stimulating *N*-butanoyl-l-homoserine lactone (C4-HSL) production^53^. Additionally, azurin expression is not regulated as a virulence factor in *P. aeruginosa*, as GacA is a major positive regulator of virulence in *P. aeruginosa*^53^. Similar to the relationship between *Burkholderia* (previously considered members of the *Pseudomonas* genus) and plants^54^, this QS-independent mechanism appears to play important roles in the *P. aeruginosa*-cancer interaction. Additional studies need to be carried out to conclusively determine a mechanism of sensing in *P. aeruginosa*.

### *P. aeruginosa* is abundant in primary tumors, and its product azurin inhibits tumor growth

**Fig. 4:**
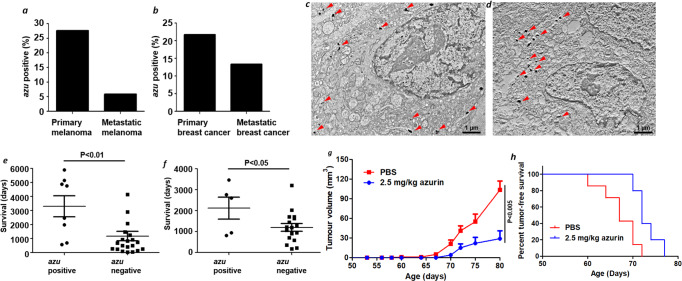
Azurin-producing *P. aeruginosa* in human tumors. The azurin-encoding gene (*azu*) was detected in tumors from patients with breast cancer and melanoma. It was amplified by PCR with *P. aeruginosa azu*-specific primers and confirmed by DNA sequencing. The amplified PCR product, as a single band, was sequenced and showed 100% identity to *P. aeruginosa azu*. In melanoma (**a**), 27.6% (8 of 29) of primary tumors and 5.9% (2 of 34) of metastatic tumors were *azu* positive (*P* < 0.05). In breast cancer (**b**), 22.7% (5 of 22) of primary tumors and 14.3% (2 of 14) of metastatic tumors were *azu* positive (*P* > 0.05). In addition, frozen tumor samples were processed for immunogold transmission electron microscopy (TEM) by using anti-*P. aeruginosa* and anti-azurin antibodies. TEM images of uranyl acetate-stained sections (arrowheads) showed the intracellular localization of *P. aeruginosa* (**c**) and its product azurin (**d**) in human melanoma sections that were *azu* positive by PCR. Magnification: ×3000. **e**, **f** Patients with azu-positive tumors displayed increased survival. Survival analysis of patients with *azu*-positive vs. *azu*-negative primary melanoma (**e**, *P* < 0.01) and primary breast cancer (**f**, *P* < 0.05) tumors. **g** Hemizygous MMTV-PyMT transgenic mice were injected with either PBS control or 2.5 mg/kg azurin (intraperitoneally, 3× weekly) for 2 months. The mean+SE values of tumor volume were calculated. **h** Tumor-free survival curve was generated with the PBS control and 2.5 mg/kg azurin groups of MMTV-PyMT transgenic mice. Median tumor-free survival of the PBS control and azurin-treatment groups was 67 and 72 days, respectively. *P* = 0.015.

Our in vitro results indicated that *P. aeruginosa* and cancer cells communicate with each other through the secreted proteins azurin and aldolase A, leading to the localization of *P. aeruginosa* on cancer cells (Figs. 1–3). This effect might be applicable to clinical settings. To measure *P. aeruginosa* localization in human melanoma and breast tumors, PCR with *azu*-specific primers was carried out. We included both primary and metastatic tumors, as their different characteristics may affect *P. aeruginosa* localization^55^. Among tumors from patients with melanoma (primary: *N* = 29, age range = 21–84 years old; metastatic: *N* = 34, age range = 24–85 years old) and breast cancer (primary: *N* = 22, age range = 30–81 years old; metastatic: *N* = 14, age range = 28–79 years old) (Supplementary Fig. 10), the *P. aeruginosa azu* gene was detectable in 27.6% of primary melanomas and 5.9% of metastatic melanomas (Fig. 4a). Primary breast cancers showed a higher positive rate for the *azu* gene than metastatic breast tumors (22.7% vs. 14.3%, Fig. 4b). The presence of *P. aeruginosa* and its product azurin was further confirmed by transmission electron microscopy (TEM) within *azu*-positive melanoma cells stained by an anti-*P. aeruginosa* antibody or anti-azurin antibody (Fig. 4c, d, Supplementary Fig. 11a–c). *P. aeruginosa* cells were found in the cytoplasm, and azurin was localized in both the cytoplasm and nucleus. This finding is consistent with those of our preclinical studies showing that azurin localizes in the nucleus^1,3^. These results indicate that *P. aeruginosa* was detectable in human tumors, raising the possibility that, in some individuals, azurin-producing *P. aeruginosa* may affect the biological activity of tumors through bacterial-cancer interactions. To test this possibility, we compared survival between patients with *azu*-positive and those with *azu*-negative primary melanoma and breast cancer tumors. In both melanoma (Fig. 4e) and breast cancer (Fig. 4f), patients with *azu*-positive tumors had better overall survival times than patients with *azu*-negative tumors, suggesting that *P. aeruginosa* azurin positively affects the prognosis of patients, at least for those with melanoma or breast cancer. Based on these findings, we intend to design clinical studies, which are needed to examine whether the presence of azurin-expressing *P. aeruginosa* is an important factor for patient prognosis and can be used as a biomarker.

In this study, we used a transgenic mouse model that spontaneously develops mammary tumors to investigate the impact of *P. aeruginosa* azurin on cancer in vivo. Based on the critical role of azurin in *P. aeruginosa*–cancer interactions (Figs. 1–3), purified *P. aeruginosa* azurin was injected into transgenic mice that spontaneously develop mammary tumors^56,57^. An in vitro experiment indicated that *P. aeruginosa* azurin secretion was significantly higher in the presence of triple-negative MDA-MB-231 cells than in the presence of T-47D(ER+, PR+, Her2−) cells (Supplementary Fig. 12); thus, triple-negative, p53wt MMTV-PyMT transgenic mice were used. When these mice were exposed to azurin, NIR dye-conjugated azurin preferentially localized to spontaneously developed mammary tumors (Supplementary Fig. 13a). Tumor growth and tumor weight were significantly inhibited without affecting body weight (Fig. 4g, Supplementary Fig. 13b, c), and tumor-free survival was significantly extended in the azurin-treatment group (Fig. 4h). To the best of our knowledge, these findings suggest a new role for the bacterium *P. aeruginosa* in host defense via azurin production.

## Discussion

In this study, we demonstrated the bidirectional regulation of bacterial-cancer communication in relation to the potential for *P. aeruginosa*-secreted azurin to inhibit tumor growth. Human cancer cells upregulated aldolase A secretion in response to increasing proximity to *P. aeruginosa*/azurin, which correlated with enhanced *P. aeruginosa* adherence to the cancer cells. Our results also show that cancer patients had detectable *P. aeruginosa* and azurin in their tumors and exhibited increased overall survival when they did. Finally, our results suggest host–bacterial symbiotic mutualism acting as a diverse adjunct to the host defense system on tumor homeostasis.

Increasing evidence suggests that this symbiotic host-microbiome relationship is dependent on the nature of microorganism-host interactions. For example, infectious agents cause ~20% of cancers worldwide^58^. *Helicobacter pylori* colonizes ~50% of people worldwide^59^ and cause ~90% of gastric cancers^58^. On the other hand, *H. pylori* colonization can benefit human health, because decolonization increases the risk of severe gastroesophageal reflux disease^60^. The relationship of *Pseudomonas* spp. with plants has been extensively studied. Plants develop in a microbe-rich environment and interact with microorganisms, such as *Pseudomonas* spp. (e.g., *P*. *aeruginosa*, *P*.* fluorescens* and *P. syringae*). Some bacteria, including *Pseudomonas* spp., can be either beneficial or pathogenic^61,62^. Collectively, this evidence suggests that some bacteria might be double-faced microbes in a symbiotic relationship that can both harm (parasitism) and benefit (mutualism) the host. It is still poorly understood exactly how host–microbe relationships were initiated; however, a previous report revealed that mutualism in proteobacteria, including *Pseudomonas*, has evolved independently between 34 and 39 times, implying that this arrangement is evolutionarily favored^63^. Those findings also indicate that in bacteria, mutualism has arisen most often in species that were originally parasites and pathogens^63,64^. Taken together, these results suggest that through evolution, *P. aeruginosa* has acquired abilities to both harm (parasitism) and benefit human health (mutualism) and adjusts its behavior depending on physiological/cellular conditions that can change depending on disease status^61^. Our results suggest that *P. aeruginosa* can have a symbiotic relationship with humans characterized by both mutualism and parasitism. In its parasitic relationship, *P. aeruginosa*, an opportunistic pathogen, increases morbidity and mortality in patients with CF, while in its mutualistic relationship, *P. aeruginosa* can benefit human health by suppressing^65^ and attacking^1,3,4^ malignant cells through secretion of the bacterial protein azurin in hosts harboring malignant cells (Supplementary Fig. 14).

*P. aeruginosa*, a common gram-negative, rod-shaped bacterium, can be found in numerous moist sites in soils and plants and in a wide variety of aquatic environments but also, as an opportunistic pathogen, causes serious infection under certain circumstances (e.g., in hospitalized patients with burns or CF adults with multi-resistant pseudomonas)^66^. Infection with *P. aeruginosa* continues to be a threat to cancer patients, particularly when patients have weakened immune activity due to cancer treatment. In contrast, *P. aeruginosa*, as a potential commensal bacterium, can be found in the skin of some healthy individuals and has been isolated from the throat (5%) and stool (3%) of non-hospitalized people^67^. In our study, *P. aeruginosa* expressing *azu* was found in tumors of primary melanoma and breast cancer patients who did not receive chemotherapy before specimen collection. Furthermore, our preliminary data on human tumors indicated that *azu*-positive patients had longer overall survival times than *azu*-negative patients, suggesting that *P. aeruginosa* localized in tumors may positively influence cancer prognosis. This possibility needs to be investigated in larger-scale clinical studies.

The bacteria-based strategies have great promise in the treatment of tumors^68^. Various bacterial species including *P. aeruginosa* showed potent anti-tumor efficacy with or without modulating immune activity^69^. A recent report suggested that factors secreted from *P. aeruginosa* altered the proliferation of breast cancer cells and enhanced the activity of doxorubicin, suggesting that the signaling stimulated by factors secreted from *P. aeruginosa*is dependent on the abundance of *P. aeruginosa*^70^. This evidence was consistent with our data that azurin secretion by *P. aeruginosa* is population- and distance-dependent (Figs. 1, 3). Furthermore, several proteins from *P. aeruginosa* such as azurin, exotoxin A (ExoA), exoenzyme T (ExoT), Pacaspase recruitment domain (Pa-CARD) have been found to exert potent cytotoxicity against cancer^69^. Moreover, azurin can enhance the efficacy of standard chemotherapeutic agents such as 5-fluorouracil (5-FU)^71^. Since *P. aeruginosa* also produces numerous virulence factors^49^, it is logical to design potential anticancer agents based on the cupredoxin family of proteins^30–32^. As we previously reported, a peptide fragment identified in azurin (a.k.a. p28) enhances the activity of doxorubicin though activation of the p53-p21 axis^29^.

In conclusion, we attempted to understand the interaction of *P. aeruginosa* with human cancer cells and its role in tumor homeostasis. We demonstrated that molecular determinants of host–bacterial mutualism act as diverse adjuncts to the host defense system through inter-kingdom signaling by evolutionarily conserved proteins, the bacterial cupredoxin azurin and human aldolase A. To the best of our knowledge, these data on a host–microbe interaction provided novel insight into the symbiotic strategies based on the microbial cupredoxin azurin in modulating host defense mechanisms, which occurred independent of immune system stimulation. Our results highlight the importance of the bacteria-cancer interaction and will be helpful for guiding further studies to understand the differences between pathogens and beneficial microorganisms. Since evidence that microbes play an important role in establishing host homeostasis (*e.g*., tumor regression) is increasing, a better understanding of the symbiotic relationship of such bacteria with humans offers enormous potential to develop therapeutic strategies for human diseases such as cancer.

## Methods

### *P. aeruginosa* and human cell lines

*P. aeruginosa* strain 8822 isolated from the sputum of a CF patient is a generous gift from Dr. Ananda M. Chakrabarty^72^. Human cell lines of prostate cancer (DU-145), normal prostate (CRL-11611), ovarian cancer (SK-OV3), normal ovary (HOSE6-3); breast cancer (MCF-7, T-47D, MDA-MB-231), normal breast (MCF-10A) were purchased from the American Tissue Culture Collection (ATCC, VA). Human melanoma (UISO-Mel-2) and CMN cell lines were developed in our laboratory as described^73,74^.

### ELISA

CF subjects were patients followed at the Adult CF Clinic at Emory University who had signed informed consent to provide blood samples in accordance with the Emory University IRB (Emory #00042577). Control sera from no-disease volunteers were obtained from Discovery Life Sciences (Los Osos, CA). Serum samples (*N* = 50 in each group) in carbonate bicarbonate buffer (pH 9.4) were used to coat 96-well plates (MaxiSorb, Thermo Fisher) in triplicate. Standard curves as internal controls were generated by using purified azurin^75^ to coat 96-well plates. Polyclonal rabbit anti-azurin antibody^1,2^ and alkaline phosphatase-conjugated secondary anti-rabbit antibody (SigmaMillipore) were used to detect azurin.

### Growth media

All human cell lines were maintained in MEME with 10% FBS except for MCF-10A which was maintained in DMEM with 10% FBS. Luria-Bertani medium was used to grow *P. aeruginosa* before transfer to the experiment’s medium of 0.5% Minimal Glucose Medium (MGM) without any antibiotics.

### *P. aeruginosa* quantitation

This was conducted using the turbidimetry method after the establishment of the standard growth curve of *P. aeruginosa* in 0.5% MGM. An optic density (OD) of 0.3 correlated with the mid-log phase of the bacterial growth and was chosen as the standard OD at 600 nm for the entire experiment.

Expression and purification of azurin: Cloning, expression of azu gene in *E. coli* and purification of recombinant azurin were described previously^1^.

### *P. aeruginosa* and human cancer cells co-incubation

*P. aeruginosa* strain 8822 were grown overnight in LB medium at 37 °C then transferred at 1:100 v:v into 0.5% MGM without adding antibiotics. The OD of the medium was measured until the goal OD for the experiment was reached. Human cells were grown in the appropriate medium as reported above. Cells were trypsinized and counted using a Coulter Counter^®^ Cell and Particle Analyzer. The needed number of cells was washed 2× with PBS and finally suspended in 0.5% MGM. For direct co-incubation assays, human cells and *P. aeruginosa* were co-incubated for 30 min at a concentration of 500,000 human cells/ml and *P. aeruginosa* at an optical density (OD) = 0.3. The needed number of cells was transferred based on the specifics of each experiment. For the indirect co-incubation assays, Corning Transwell^®^ polyester membrane cell culture plates and inserts (TC-treated, sterile 24 mm Transwell with 0.4 µm pores, SigmaMillipore) were used. Human cells were immobilized in the lower compartment (well) whereas bacteria were incubated in the upper compartment (insert) at 37 °C for 30 min with a semi-permeable membrane of 0.4 µm pores separating the two compartments. Wild type (WT) *P. aeruginosa* PAO1 and its azu^null^ mutant cells were used for aldolase secretion assay. Mutant *P. aeruginosa* was maintained on LB agar plates containing spectinomycin^37^. Other experimental conditions were the same as above.

### Protein extraction

Comparative analyses of secreted proteins in media were performed as described previously^76–78^. The same volume of medium was collected from cell suspensions (direct co-incubation assays) or the lower compartment well (indirect co-incubation assays) after incubation and centrifuged at high speed (12,000 × *g*). The supernatant was then filtered using an Amicon^®^ 0.22 µm pore filter. The equal volume of trichloroacetic acid (TCA; final concentration of 20%) was added to the supernatant and incubated on ice for 30 min. After spinning at 18,000 × *g*, TCA was decanted, and 100% acetone precipitation was applied twice with 5 min incubation. At the final step, acetone was decanted, and protein pellets were allowed to dry at room temperature for 15 min and resuspended in PBS. The equal volume of each sample was loaded per lane.

### Western blotting

After running the NuPAGE, proteins were transferred to PVDF membranes (BioRad Laboratories Inc, Hercules, CA) which were blocked in SuperBlock (T20 TBS buffer, Thermo Fisher) for one hour. For secreted proteins, equal loading was confirmed by Ponceau staining of the membranes^79^. The PVDF membranes were incubated in a polyclonal rabbit anti-azurin antibody^1^ (1:5000) or a monoclonal mouse anti-aldolase A (1:200, Santa Cruz Biotechnology, TX) at 4 °C overnight. For cell lysates, GAPDH was used as a loading control (Novus Biologicals). The secondary antibody was applied (1:1000, polyclonal goat anti-rabbit IgG-HRP; Santa Cruz Biotechnology). The signal was detected using enhanced chemiluminescence (ECL, Thermo Fisher Scientific). Quantitative analysis of the bands was performed with a computer-assisted imaging densitometer (UN-SCAN-IT gel version 5.1).

### Host cell adhesion assay

The assays were performed essentially as described before^38^. Cancer cells were co-incubated with *P. aeruginosa* 8822 in 96-well plates in the presence or absence of various concentrations of aldolase A for 2 h. Monolayers of cancer cells were extensively washed with PBS to remove unbound *P. aeruginosa*. Monolayers were homogenized in 0.1% saponin (SigmaMillipore) in PBS. *P. aeruginosa* were enumerated by serial dilution of the homogenized suspensions and subsequent determination of colony-forming units (CFU/ml) by plating on LB agar plates. Control values of CFU/ml expressed as 100%. siRNA-induced silencing of aldolase and MUC1 genes in MDA-MB-231 and Mel2 cells as conducted as described previously^80,81^. Briefly, SMARTpool human ALDOA, MUC1, and non-targeting siRNA pool (non-targeting siRNAs) (Dharmacon, PA) were used as siRNA targeting aldolase A, MUC1, and negative control, respectively. MDA-MB-231 and Mel2 cells were seeded in 96-well plates, cultured to 80–90% confluence, transfected with 120 nM siRNAs using FuGENE HD (Promega) according to the manufacturer’s protocol for 48 h, and used for host cell adhesion assays. Knockdown of ALDOA and MUC1 by siRNA transfection was examined by western blot analyses using monoclonal mouse anti-aldolase A and MUC1 antibodies (1:200, Santa Cruz Biotechnology).

### Detection of azurin gene in human tumors

Tumor samples of breast cancer and melanoma were collected at University of Illinois at Chicago. All patients included in the analysis were diagnosed with either breast cancer or melanoma. Supplementary Fig. 10 contains relevant patient information with similar age range and median age. The detection of *azu* gene in the human tumors was performed by PCR with *azu*-specific primers; 5′-CAGTTCACCGTCAACCTGTCC-3′ and 5′-TGGTGTGGGCGATGACACG-3′. Human GAPDH was also amplified as a loading control; 5′-AACGGGAAG CTTGTCATCAA-3′ and 5′-TGGACTCCACGACGTACTCA-3′. We used ultraclean instruments, kits, and reagents to minimize and control for contamination. DNeasy Blood & Tissue Kits (Qiagen) were used to prepare template DNA. The amplified PCR products were confirmed as a single band on 2% agarose gels. Products were purified by using QIAquick PCR Purification kits (Qiagen) and sequenced with an ABI Prism 3700 DNA analyzer. The Fisher’s Exact Test was applied to determine if the positive rates are significantly different between the subjects with the primary and the metastatic tumors.

### *P. aeruginosa* and azurin visualization by TEM

Frozen tumors were processed for immunogold transmission electron microscopy in accordance with a published method^82,83^ with minor modifications. Tumor samples along with their survival data were obtained from patients who had signed informed consent (IRB #H-96-772). The frozen samples were diced into 1 × 1 mm cubes on dry ice then fixed by incubating them in PBS-containing 4% paraformaldehyde and 0.75% glutaraldehyde for 72 h at 4 °C. The samples were thoroughly washed with PBS then were incubated with PBS-containing 0.1% saponin for 1 h at room temperature on a rotary mixer (Ted Pella, Inc, Redding, CA). After the samples were thoroughly washed with PBS, they were incubated with PBS-containing 5% bovine serum albumin (BSA), 0.1% cold water fish skin gelatin (CWFS), and 5% normal goat serum (NGS) (Goat Gold Conjugate Blocking Solution, Electron Microscopy Sciences, Hatfield, PA) for 1 h at 4 °C. The samples were thoroughly washed with PBS-containing 0.1% acetylated bovine serum albumin (BSA-c) (Aurion BSA-c, Electron Microscopy Sciences) then incubated with either mouse anti-*Pseudomonas aeruginosa* antibody (clone B11, 1:50 dilution, Thermo Fisher Scientific, Waltham, MA) or rabbit anti-azurin antibody (1:100 dilution)^1^ for 48 h at 4 °C. After extensive washing, the samples were incubated with 0.1% BSA-c/PBS-containing either goat anti-mouse or goat anti-rabbit secondary antibody for 24 h at 4 °C. To visualize each specific primary antibody, these antibodies were labeled with 10 nm gold particles (1:50 dilution, Electron Microscopy Sciences). The samples were washed with PBS-0.1% BSA-c and deionized water then incubated with deionized water containing 2.5% glutaraldehyde (Electron Microscopy Sciences) for 1 h at room temperature. After extensive washing with deionized water, the sections were fixed with deionized water containing 0.5% osmium tetroxide and 1.5% potassium ferricyanide (Electron Microscopy Sciences) for 15 min in the dark. Next, the samples were dehydrated by incubation in an ascending series of ethanol (25, 50, 75, 95, 100%, Electron Microscopy Sciences) followed by incubation in a 1 to 1 ratio of 100% ethanol to epoxy resin (comprised of a mixture of EMbed 812, nadic methyl anhydride, dodecenyl succinic anhydride, and 2,4,6-Tris(dimethylaminomethyl)phenol, Electron Microscopy Sciences) for 12 h at room temperature on a rotary mixture (Ted Pella, Inc). The samples were incubated with 100% epoxy resin for 12 h at room temperature on a rotary mixer (Ted Pella, Inc). The epoxy resin was changed and the samples were incubated for 2 h at room temperature on a rotary mixer. The samples were placed into flat embedding molds filled with epoxy resin then allowed to polymerize at 70 °C for 24 h. Ultrathin sections (70 nm) were cut with an ultramicrotome (EM UC7, Leica Microsystems, Buffalo Grove, IL), mounted on formvar- and carbon-coated 200 mesh copper grids (Electron Microscopy Sciences) then stained with filtered 1% uranyl acetate prior to imaging. Samples were imaged with a Philips CM 120 transmission electron microscope (TSS Microscopy, Hillsboro, OR) equipped with a BioSprint sixteen-megapixel digital camera (Advanced Microscopy Techniques, Woburn, MA).

### Patients’ survival

Survival of each patient of primary melanoma and breast cancer was measured from the date of diagnosis until death from any cause or last follow-up. The experiments were based on randomized trials and the investigators were blinded to the assignments in the experiments and outcome evaluations. Two independent sample tests were performed for comparing the survival days of *azu* gene positive and negative groups. The Shapiro–Wilk’s test showed that normality was severely violated in data of melanoma patients. The Wilcoxon’s rank sum test for the medians in the melanoma survival data was chosen due to its robustness to the deviation from normality. For breast cancer patients, Shapiro–Wilk’s test showed that the survival days in both *azu* gene positive and negative groups can be well-modeled by a normal distribution. As equal variances between the two groups were reasonable and was confirmed by a *F* test, a pooled *t* test on means was performed to compare the survival days between *azu* gene positive and negative groups. Data were analyzed using R (v.4.0.2) and RStudio (v.1.3.1093).

### Effect of azurin on transgenic mice

Hemizygous MMTV-PyMT (mouse mammary tumor virus-polyoma middle tumor-antigen) female mice were obtained from The Jackson Laboratory. Since MMTV-PyMT mice develop spontaneous mammary tumors that closely resemble the progression and morphology of human breast cancers, the mouse model is widely used and well characterized^84^. Mice at 4-weeks old were randomized into control (*N* = 7) and azurin-treatment (*N* = 5) groups. Control animals received PBS and azurin-treatment animals received 2.5 mg/kg of azurin in sterile PBS i.p. 3× week for nearly two months (80 days old). Based on azurin levels in CF patients’ sera, the highest level of azurin was 32 µg/ml. In order to reach this level of azurin in mouse (25 g-body weight, 2 ml total blood volume), azurin does at 2.5 mg/kg was determined. Tumor volume and body weights were determined three times weekly. All tumors were excised and weighted at the end of the study. All experiments were approved by University of Illinois at Chicago (UIC) Institutional Animal Care and Use Committee (IACUC) and conformed to the guidelines set by United States Animal Welfare Act and the National Institutes of Health.

### Statistics and reproducibility

The paired student’s *t* test the one-way analysis of variance were used for comparisons. *P* value < 0.05 was considered significant. Number of replicates (*N*) can be found in the figure legends. Data were analyzed using Graphpad Prism software (ver. 8), R (ver.4.0.2), and RStudio (ver.1.3.1093).

### Reporting summary

Further information on research design is available in the Nature Portfolio Reporting Summary linked to this article.

## Supplementary information

Supplementary Information

Reporting Summary

## Data Availability

The data that support the findings of this study are available in the supplementary information. Also, the datasets presented in the main figures are deposited in the repository Figshare; 10.6084/m9.figshare.21644321, 10.6084/m9.figshare.21644333, 10.6084/m9.figshare.21644336, 10.6084/m9.figshare.21644342, 10.6084/m9.figshare.21644345, 10.6084/m9.figshare.21644348, and 10.6084/m9.figshare.21644351. No computer code is involved in this study. Uncropped images for blots are available in the supplementary information.
