# Supplementary material for: Cross-talk between cancer and Pseudomonas aeruginosa mediates tumor suppression
Source: Commun Biol. 2023 Jan 6;6:16. doi: 10.1038/s42003-022-04395-5 (PMC9823004; doi:10.1038/s42003-022-04395-5)

Supplementary Information

Cross-talk between cancer and *Pseudomonas aeruginosa* mediates tumour suppression

Juliana K. Choi[1,2,#], Samer A. Naffouje[1,3,#], Masahide Goto[1], Jing Wang[4], Konstantin Christov[1], David J. Rademacher[5], Albert Green[1], Arlene A. Stecenko[6], Ananda M. Chakrabarty[7], Tapas K. Das Gupta[1,*] and Tohru Yamada[1,8,*]

[1] Department of Surgery, Division of Surgical Oncology, University of Illinois College of Medicine, Chicago, IL 60612, USA

[2] Current address: Department of Dermatology, University of Pennsylvania Perelman School of Medicine, Philadelphia, PA 19104, USA

[3] Current address: General Surgery, Cleveland Clinic, Cleveland, OH 44195, USA

[4] Department of Mathematics, Statistics and Computer Science, University of Illinois College of Liberal Arts and Sciences, Chicago, IL 60612, USA

[5] Department of Microbiology and Immunology and Core Imaging Facility, Loyola University Chicago, Maywood, IL 60153, USA

[6] Division of Pulmonary, Asthma, Cystic Fibrosis, and Sleep, Department of Pediatrics, Emory University School of Medicine, Atlanta, GA 30322, USA

[7] Department of Microbiology & Immunology, University of Illinois College of Medicine, Chicago, IL 60612, USA

[8] Richard & Loan Hill Department of Biomedical Engineering, University of Illinois College of Engineering, Chicago, IL 60607, USA

# These authors contributed equally: Juliana K. Choi and Samer A. Naffouje

* Corresponding authors: tkdg@uic.edu and tohru@uic.edu

Methods for Supplementary Data

RNA extraction and real-time PCR for azurin expression: RT-PCR was performed with 1 µg of total RNA from control and treated samples using the SuperScript First-Strand Synthesis System for real-time reverse transcriptase polymerase chain reaction (RT-PCR) (Invitrogen, Carlsbad, CA) and SYBR® Green PCR Master Mix (Applied Biosystems, Foster City, CA). Software from Integrated DNA Technologies (Coralville, IA) was used to design primers. The reaction was conducted as a two-step RT-PCR procedure using RT-PCR reagents kits from Invitrogen, and ABI 7500FAST Sequence Detection System (Applied Biosystems, Foster City, CA). After the last amplification cycle, the PCR products were denatured to generate a dissociation curve. The fluorescent signal was detected and analyzed by ABI Prism® 7500 Sequence Detection Software (Applied Biosystems, Foster City, CA). The threshold cycles (Ct) were calculated by the software and used to determine relative expression of genes.

Protein identification by Mass spectrometry: Mel-2 and *P. aeruginosa* 8822 were co-incubated using the Transwell system and secretions were concentrated using Amicon Centriprep YM-3 centrifugal filters (Thermo Fisher Scientific). The concentrated samples were run on SDS-PAGE. The gels were stained with Coomassie Brilliant Blue R-250 (CBB) (0.1% CBB R-250, 20% methanol, 0.5% acetic acid) and destained for an hour (BioRad). The Mel-2 and P. aeruginosa sample demonstrated an additional band when compared to a Mel-2 only sample. The additional protein band was cut from the gel. In-gel tryptic digestion and matrix-assisted laser desorption/ionization time-of-flight (MALDI-TOF) mass spectrometry of tryptic peptides was performed as follows. Gel plugs were washed in 50% acetonitrile, reduced of sulfide bonds in 60 mM DTT, alkylated of free sulfhydryl groups in iodoacetamide, 50 mM ammonium bicarbonate (pH 8.0) and 5 mM EDTA, and then incubated in trypsin [in 50 mM ammonium bicarbonate (pH8.0) solution at a concentration of 2 µg/100 µl] overnight. For MALDI-TOF, residual peptides were extracted, spotted onto a MALDI-TOF target, and analyzed by a positive-ion reflector mode with delayed extraction over the m/z range 700–4000 using a Voyager DE-PRO Mass Spectrometer (Applied Biosystems, Foster City, CA) equipped with a nitrogen laser. Spectra were externally and internally calibrated. Peptide mass results were used to identify the proteins using the MASCOT Peptide Fingerprint link.

Cell count and MTT assays: Growth of Mel-2 cells after co-incubation with *P. aeruginosa* 8822 was assessed by counting the number of cells at the desired time point and comparing the numbers to the initial cell count. The cells were plated in 24-well cell culture plates at a density of 4 x 10^5 cells/well in MEME supplemented with FBS, L-glutamine and non-essential amino acids. They were incubated at 37 °C in a 5% CO_2 humidified atmosphere and allowed to adhere overnight. *P. aeruginosa* (OD=0.3) was co-cultured with Mel-2 for 30 minutes. Following the incubation, *P. aeruginosa* was aspirated, Mel-2 were washed with PBS, trypsinized, made into a single cell suspension, diluted with Isoton II Diluent, and counted in triplicates using the Coulter Counter Cell and Particle Analyzer. The total number of cells was obtained by considering the dilution factor and the total volume of the cell suspension from each well. The potential antiproliferative effects of *P. aeruginosa* 8822 on Mel-2 viability were also evaluated using the 3-[4,5-dimethylthiaolyl]-2,5-diphenyl-tetrazolium bromide (MTT) assay (TACS MTT cell proliferation assay kits, Trevigen, Gaithersburg, MD). Cells were seeded in 96 well plates at a density of 5,000 cells/well. Cell viability was analyzed after co-incubation with *P. aeruginosa* for 30 minutes. Following co-incubation with *P. aeruginosa*, Mel-2 were incubated with MTT tetrazolium reagent for 2 h at 37 °C, and the absorbance of formazan was then measured at 570 nm. Each treatment was performed in triplicates, and the percent of cell growth inhibited was calculated by comparison of the absorbance readings of the control (Mel-2 only) versus Mel-2 co-incubated with *P. aeruginosa*.

Gene expression and intracellular levels of aldolase A. MDA-MB-231 and Mel-2 were treated with azurin for 30 min. Total RNA was extracted from cancer cells and cDNAs was generated by using High-Capacity cDNA Reverse Transcription Kits (Thermo Fisher Scientific). Conditions of reverse transcription were as follows: 25˚C for 10 min, 37˚C for 120 min, 85˚C for 5 min. Expression levels of aldolase A was determined by RTPCR using SYBR Green assay following the manufacturer's instructions [PowerUp SYBR Green Master Mix (Life Technologies, USA)]. The relative expression of aldolase A was calculated using the comparative Ct method. Data (N=3) was normalized with β-actin gene as a housekeeping gene. ΔCt = Ct aldose A – Ct β-actin. The changes of treatment in aldose A signal relative to the total amount of cDNA were expressed as ΔΔCt = Ct Treatment – Ct Control. Relative changes in treatment were then calculated as 2-ΔΔCt. The following primer sequences were used: Aldolase A: forward 5'-CGG GAA GGA GAA CCT G-3' and reverse 5'-GAC CGC TCG GAG TGT ACT TT-3'; and β-actin: forward 5'-ACT GGA ACG GTG AAG GTG AC-3' and reverse 5'-AGA GAA GTG GGG TGG CTT TT-3'. Real-time PCR was performed under the following conditions: 50˚C for 2 min, 95˚C for 2 min; 40 cycles at 95˚C for 15 sec and 60˚C for 1 min; and 95˚C for 15 sec, 60˚C for 1 min and 95˚C for 15 sec.

NIR-imaging of transgenic mice: Near infrared red fluorescent dye (IR800, Licor, NE) was conjugated to azurin according to the manufacturer instructions. Transgenic mice with spontaneously developed mammary tumours received NIR dye conjugated azurin at 5 mg/kg once i.p. After 24 h, specific fluorescence signal at 800 nm was recorded by the PDE-neo® NIR camera system (Hamamatsu photonics, Mitaka-USA).

a

Patient demographics				
	Number of patients	Sex	Age range (years)	Median age (years)
**CF patients**	50	Male: 25 (50%)<br>Female: 25 (50%)	18-42	26
**Healthy volunteers**	50	Male: 31 (62%)<br>Female: 19 (38%)	20-40	28

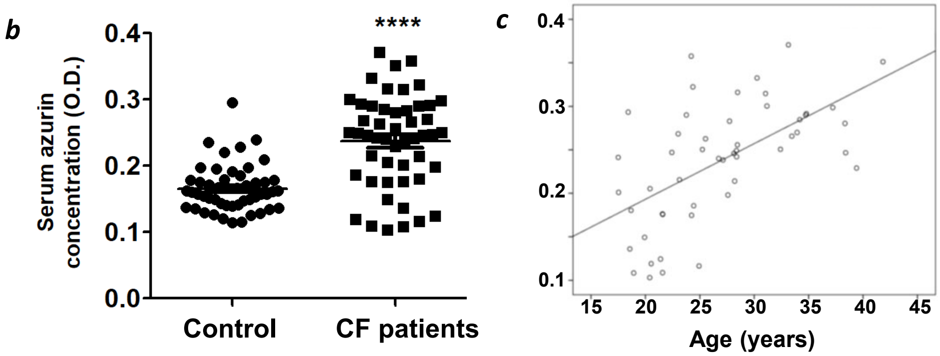

Supplementary Fig. 1: Serum azurin levels are elevated in cystic fibrosis patients. ***a***. Patient demographics. Serum from cystic fibrosis (CF) patients and controls (N=50 each) with similar age ranges/median ages was used to measure serum azurin levels. ***b***. Serum levels of azurin in CF patients (N=50) and controls (N=50) were determined by ELISA with a rabbit polyclonal anti-azurin antibody an anti-azurin antibody. For internal standards, purified azurin was used. ***c***. The linear regression line of azurin level vs. CF patient age was plotted. R=0.596, P=0.0038. Statistical analysis (t-test) showed a significant difference in azurin levels between CF patients and healthy controls. ****: P<0.0001.

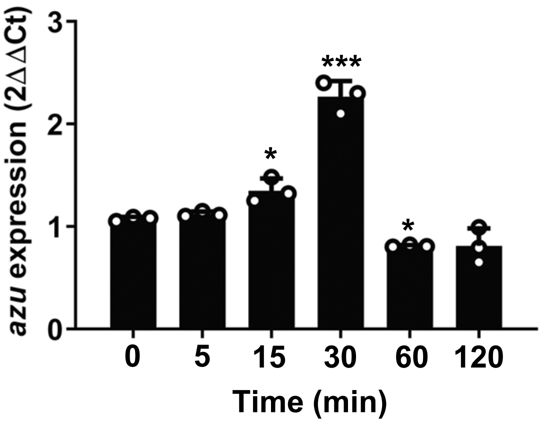

Supplementary Fig. 2: Azurin transcription is induced upon co-incubation of *P. aeruginosa* with Mel-2 cells. *P. aeruginosa* and Mel-2 cells were indirectly co-incubated for 0 to 120 min, and azu transcription was assessed by real-time PCR. Analysis of ΔΔCt values was conducted for azu transcripts with normalization to rpoD transcripts. azu and rpoD mRNA was isolated at various time points of co-incubation of *P. aeruginosa* cells with Mel-2 cells. The data revealed a 2-fold increase in the azurin transcript level at the 30-minute time point. Mean+SD, * $P<0.05$, *** $P<0.001$ (ANOVA, vs. the control (rpoD).

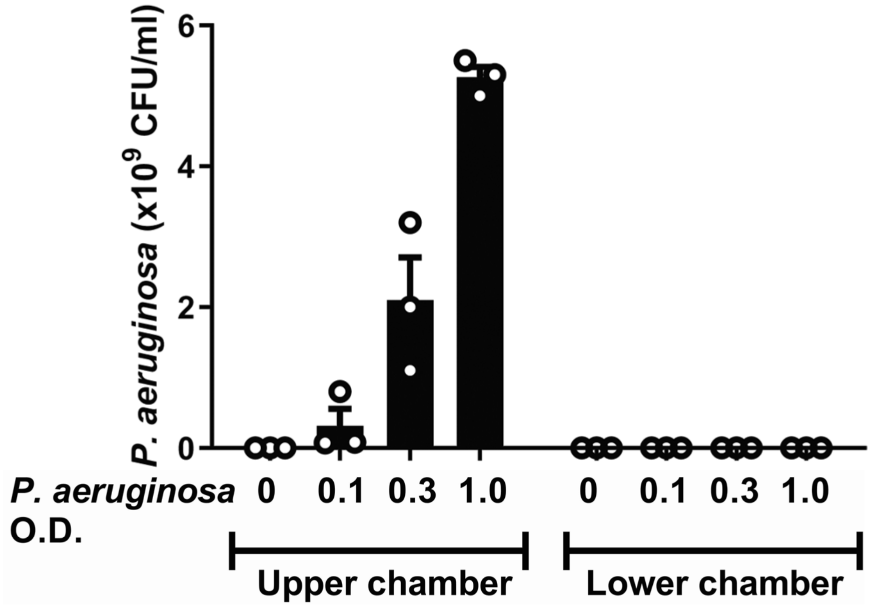

Supplementary Fig. 3: *P. aeruginosa* does not pass through the 0.4 µm filter membrane. Corning Transwell® polyester membrane cell culture plates and inserts (TC-treated, sterile 24 mm Transwell with 0.4 µm pores) were used for the assays. Various concentrations of *P. aeruginosa* in 0.5% MGM were incubated in the upper compartment (insert) at 37 ºC. After 30 min incubation, culture media from upper and lower (well) chambers were plated on LB agar plates and determined the colony-forming units (CFU/ml).

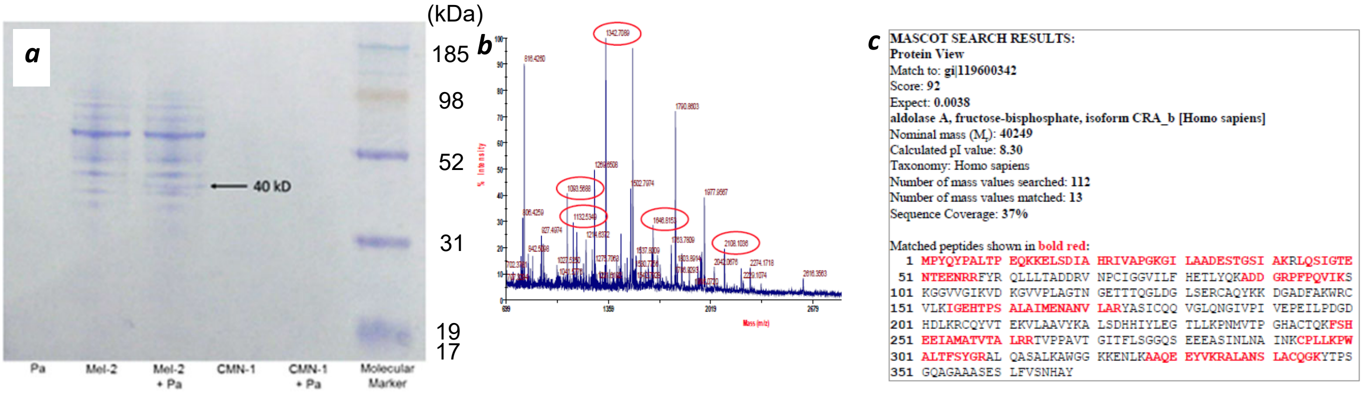

Supplementary Fig. 4: Aldolase A secretion by melanoma cells is induced by exposure to azurin. Aldolase A is secreted by Mel-2 cells in the presence of *P. aeruginosa*. ***a***. Mel-2 or CMN cells (3,000,000 cells/ml) and *P. aeruginosa* (OD=0.3) were co-incubated in the Transwell system, and culture supernatants were collected and subjected to SDS-PAGE followed by Coomassie staining. The Mel-2 + *P. aeruginosa* sample demonstrated one additional band (at 40 kDa) compared to the Mel-2 sample. ***b***. This protein band was analysed by mass spectrometry, and the protein was identified as human aldolase A. ***c***. The numbers circled in red indicate peptides that matched the aldolase A sequence. Based on the mass spectrometry data, Mascot software was used to identify protein/peptide sequences. Matched peptide sequences to human aldolase A from the mass spectrometry data are shown in red.

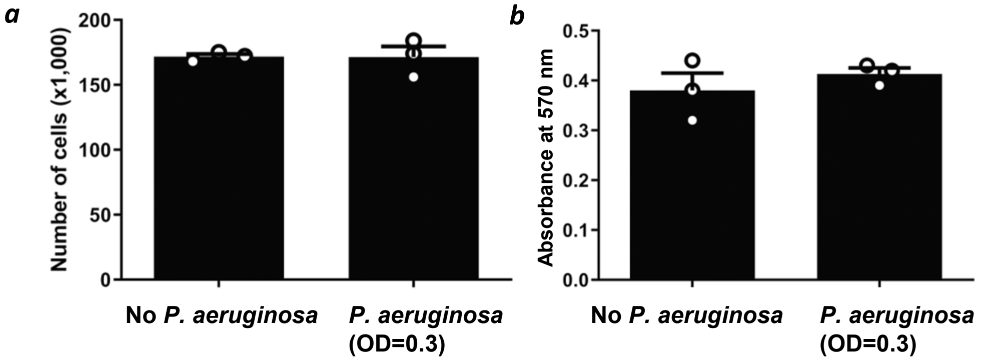

Supplementary Fig. 5: *P. aeruginosa* does not induce cytotoxicity in Mel-2 cells during co-incubation for 30 min. To confirm that the factors found in the extracellular environment of Mel-2 cells are released due to active secretion and not due to cytotoxicity, cell viability assays were conducted on Mel-2 cells co-incubated with *P. aeruginosa*. The mean+SEM values were calculated from (***a***) cell count and (***b***) MTT cell proliferation assay data, and no significant difference was observed between the viability of Mel-2 cells in monoculture or in co-culture with *P. aeruginosa*.

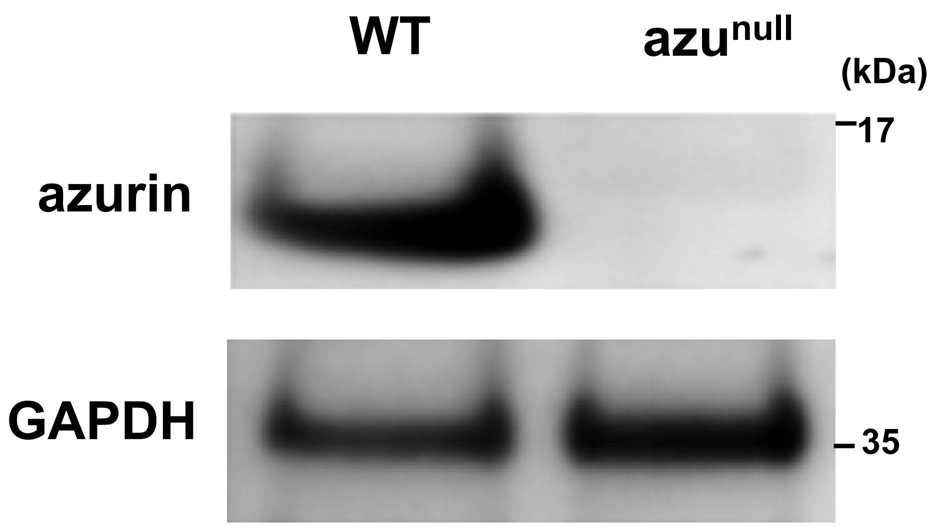

Supplementary Fig. 6: Western blot analyses of azurin in WT and mutant *P. aeruginosa*. Cell lysates of wild type (WT) and azu gene null *P. aeruginosa* PAO1 were loaded on the 4-12% NuPAGE gels. Bacterial proteins in the gel were transferred to the nitrocellulose membrane. Rabbit anti-azurin antibody (1:5,000) and anti-GAPDH (1:5,000) antibodies were applied and HRP-conjugated secondary antibodies were used for band visualization.

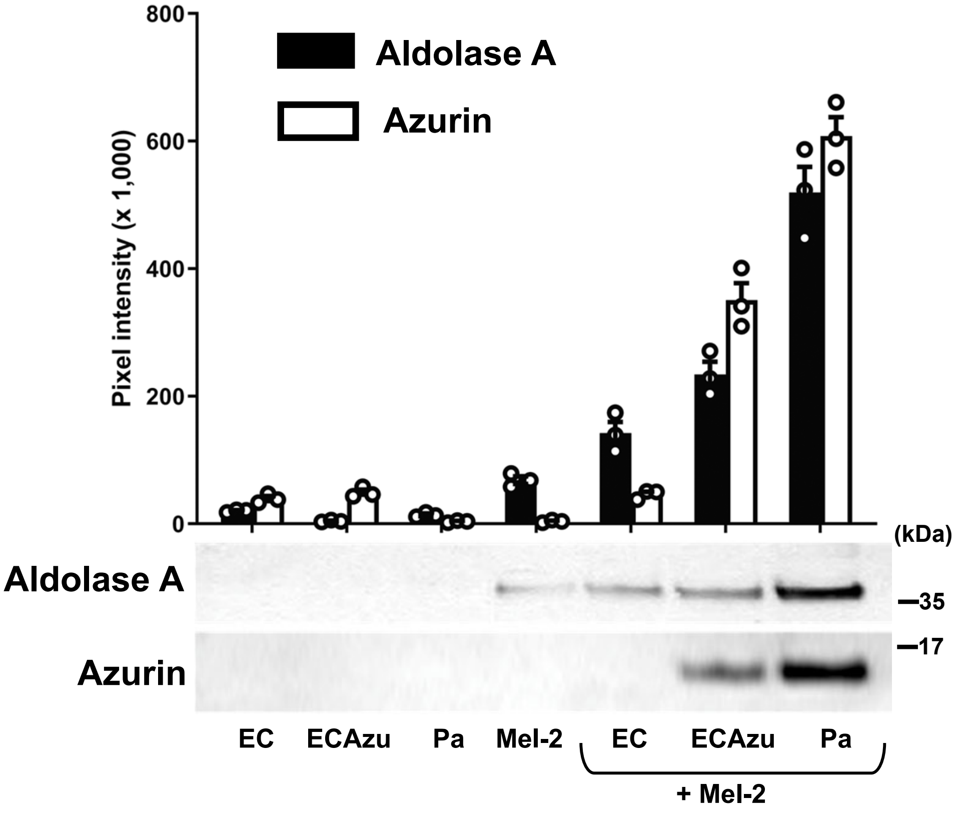

Supplementary Fig. 7: *E. coli* harbouring the *P. aeruginosa azu* gene (ECAzu) induces aldolase secretion from Mel-2 cells. Both *P. aeruginosa* (Pa) and ECAzu demonstrated azurin secretion in the presence of Mel-2 cells. However, compared to ECAzu, Pa elicited a greater than 4-fold increase in azurin secretion. *E. coli* (EC), ECAzu, and Pa stimulated aldolase secretion from Mel-2 cells. Compared to EC, ECAzu demonstrated a 2-fold increase and Pa demonstrated a 6-fold increase in aldolase secretion. Secretion of azurin and aldolase into the culture supernatant was assessed by western blot analysis. Mean+SEM.

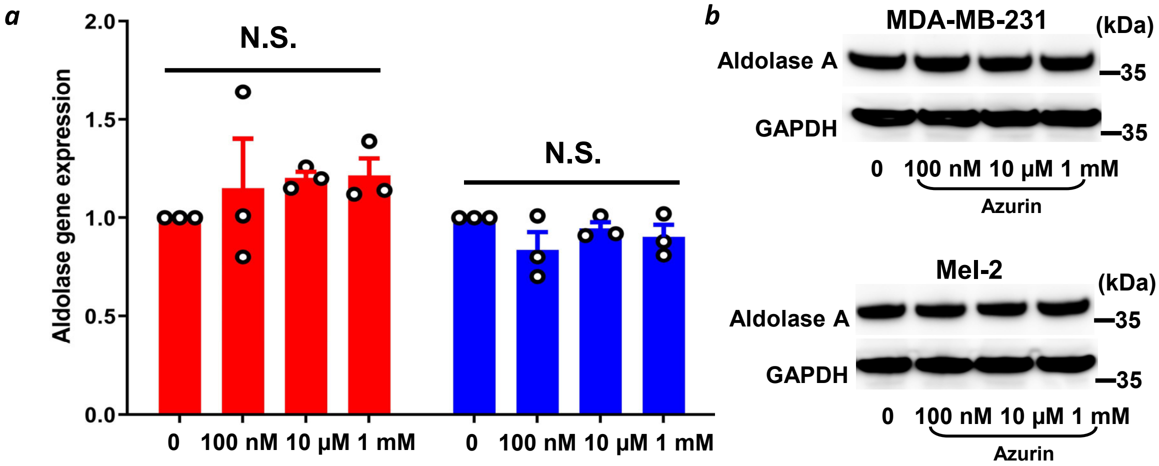

Supplementary Fig. 8: Azurin does not modulate the gene expression or intracellular levels of aldolase A. MDA-MB-231 and Mel-2 cells were treated with purified azurin protein at concentrations of 100 nM, 10 μM, and 1 mM for 30 min. Aldolase A gene expression in MDA-MB-231 (black bars) and Mel-2 (white bars) cells was determined by RT-PCR (***a***). N.S.: not significant. Mean+SEM. ***b***. Intracellular levels of aldolase A were assessed by western blot analysis with an anti-aldolase A antibody. GAPDH was used as a loading control.

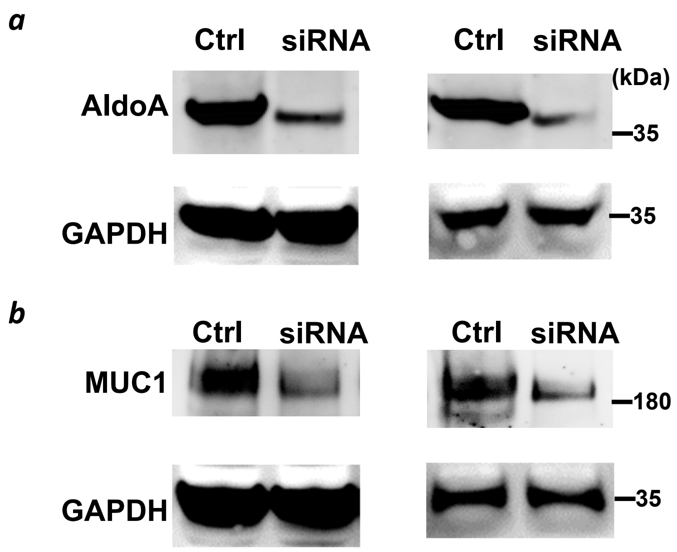

Supplementary Fig. 9: siRNA-induced silencing of aldolase A (***a***) and MUC1 (***b***) genes in MDA-MB-231 (left) and Mel2 (right) cells. SMARTpool human ALDOA, MUC1 and non-targeting siRNA pool (Ctrl) were used as siRNA targeting aldolase A, MUC1 and control (Ctrl), respectively. After 48 h of transfection, whole cell lysates (30 μg/lane) were loaded on 4-12% NuPAGE gels. Proteins in the gels were transferred to the nitrocellulose membranes. Anti-aldolase, MUC1, and anti-GAPDH antibodies were applied, and HRP-conjugated secondary antibodies were used for band visualization.

Melanoma				
	**Number of patients**	**Sex**	**Age range (years)**	**Median age (years)**
**Primary tumour**	29	Male: 19 (65 %)<br>Female: 10 (35 %)	21-84	53
**Metastatic tumour**	34	Male: 21 (62 %)<br>Female: 13 (38 %)	24-85	49

Breast cancer				
	**Number of patients**	**Sex**	**Age range (years)**	**Median age (years)**
**Primary tumour**	22	Male: 0 (0 %)<br>Female: 22 (100 %)	30-81	55
**Metastatic tumour**	14	Male: 0 (0 %)<br>Female: 14 (100 %)	28-79	51

Supplementary Fig. 10: Demographics of patients with melanoma and breast cancer. Primary and metastatic tumours from patients with similar age ranges and median ages were used.

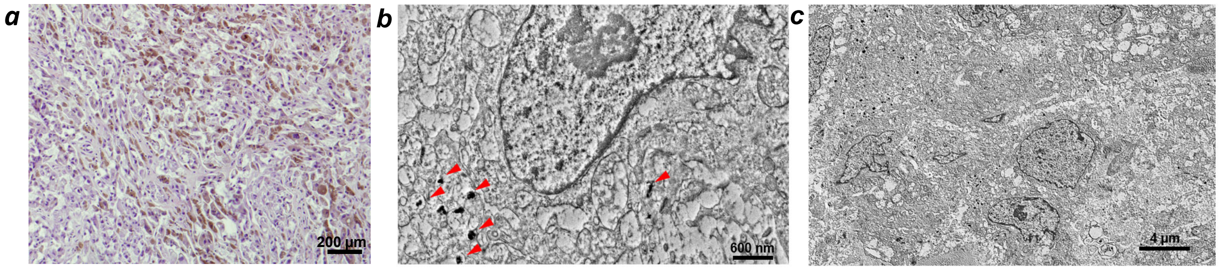

Supplementary Fig. 11: Melanoma specimen sections contain *P. aeruginosa* and azurin. ***a.*** H&E staining of human melanoma that was imaged by TEM in Fig. 4. The H&E-stained sections (20x magnification) confirmed a malignant tumour with high cellular atypia. In approximately 20% of tumour cells, the cytoplasm was stained brown due to the expression of melanin. TEM images showed the intracellular localization of azurin (***b***, 6,300x; ***c***, 1,000x) in the human melanoma sections (arrowheads).

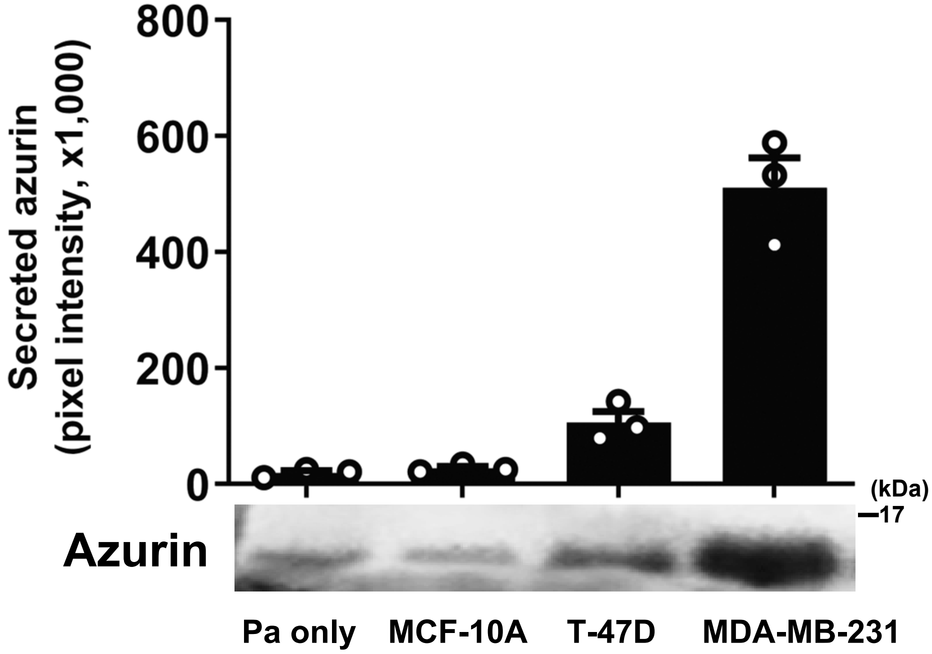

Supplementary Fig. 12: Invasive oestrogen receptor and progesterone receptor-negative breast cancer cells induce the highest level of azurin secretion by *P. aeruginosa. P. aeruginosa* secretes higher levels of azurin in the presence of highly invasive MDA-MB-231 (ER-, PR-, Her2-) human breast cancer cells than in the presence of non-invasive T-47D (ER+, PR+, Her2-) human breast cancer cells or the MCF-10A normal breast cell line. Cancer [highly invasive MDA-MB-231 (ER-, PR-, Her2-) human breast cancer cells and non-invasive T-47D (ER+, PR+, Her2-) human breast cancer cells] and normal/non-malignant cells (MCF-10A cells) were co-incubated with *P. aeruginosa* for 30 min. Azurin secretion by *P. aeruginosa* into the culture supernatant was assessed by western blot analysis; the graph shows the observed band intensities. Mean+SEM.

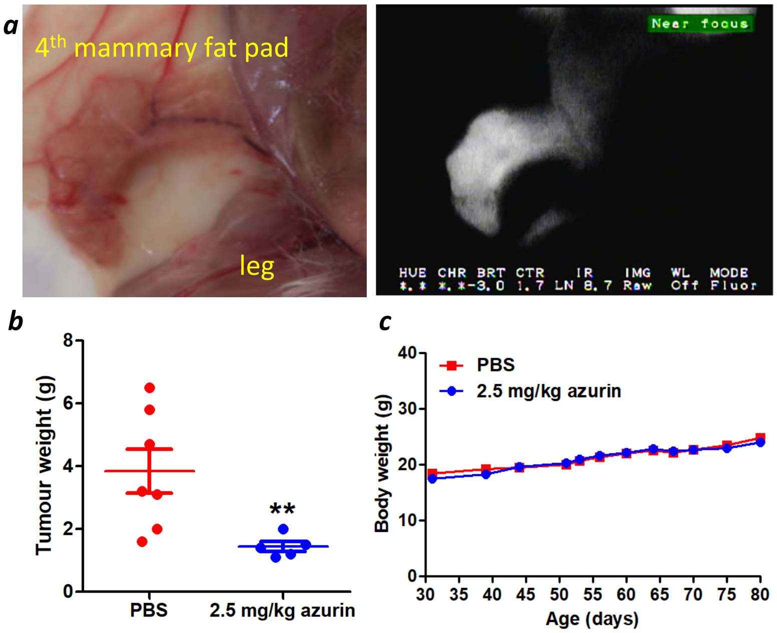

Supplementary Fig. 13: Effect of azurin from *P. aeruginosa* in a transgenic animal model. ***a.*** Near-infrared red (NIR) dye-conjugated azurin or PBS was injected i.v. into transgenic mice that spontaneously develop mammary tumours. Twenty-four hours after azurin injection, mice were imaged with a photodynamic eye (PDE) system (left: photographic image, right: NIR fluorescence image at 800 nm). ***b***. At the end of the study, the tumours were resected. The tumour weight of azurin-treated mice was significantly lower than that of control mice (** P<0.01), but the body weight did not differ (***c***).

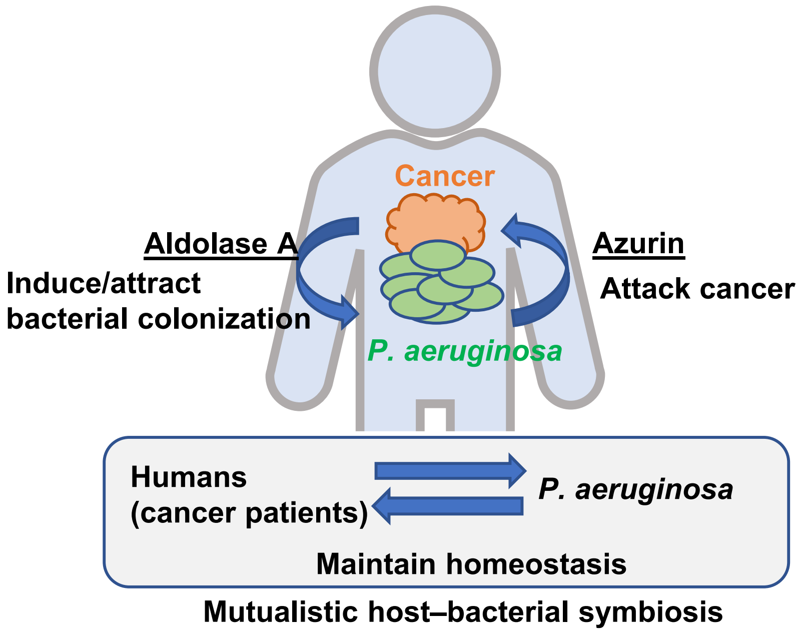

Supplementary Fig. 14: A model of the *P. aeruginosa*-cancer interaction. Aldolase A secretion in response to the bacterial protein azurin a beneficial anti-cancer activity of bacteria.

Fig 1a

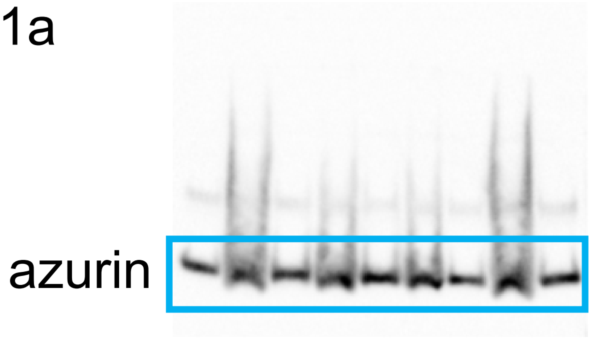

Fig 1b

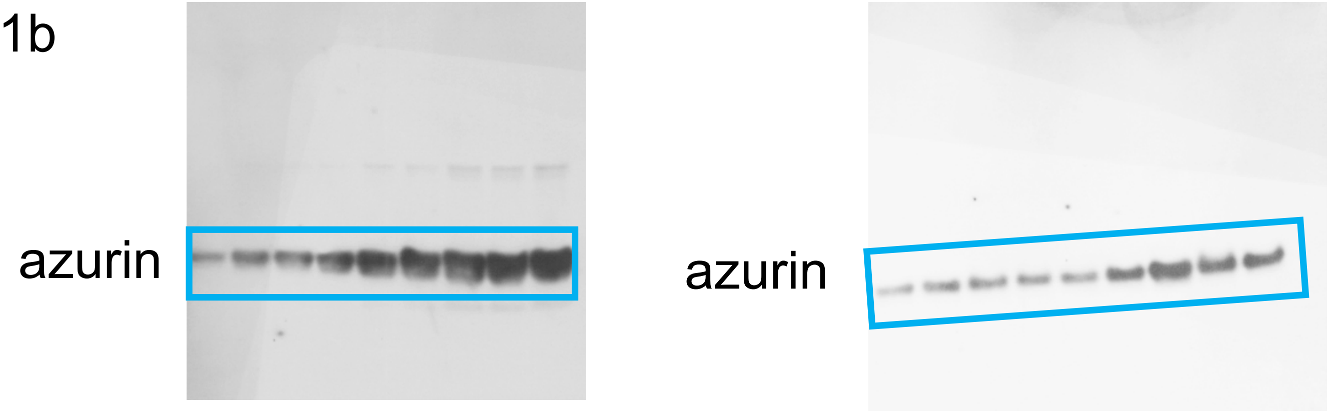

Fig 1c

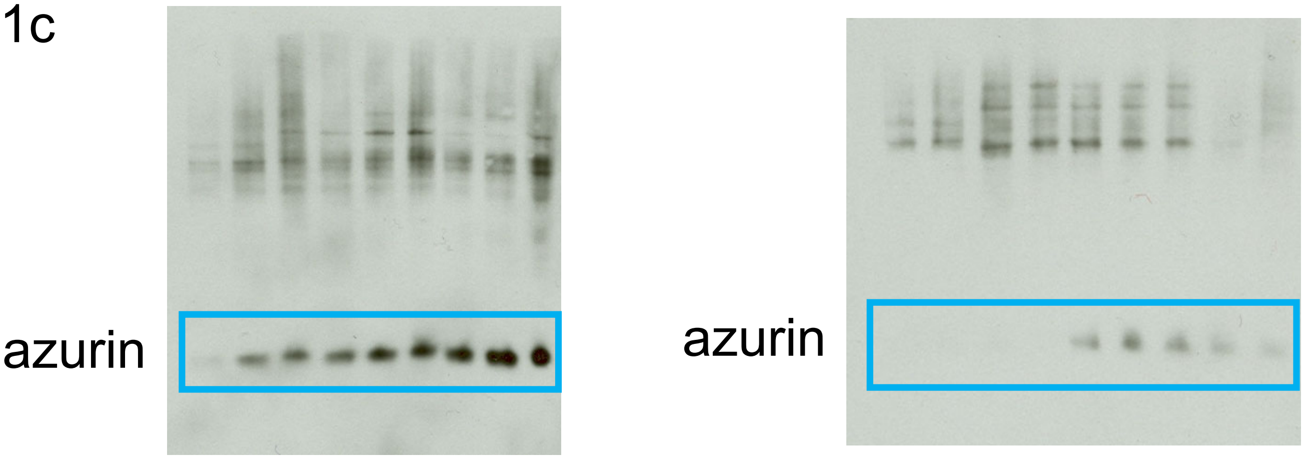

Fig 1d

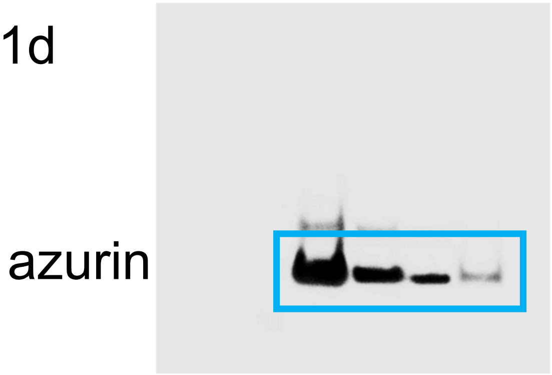

Fig 2a

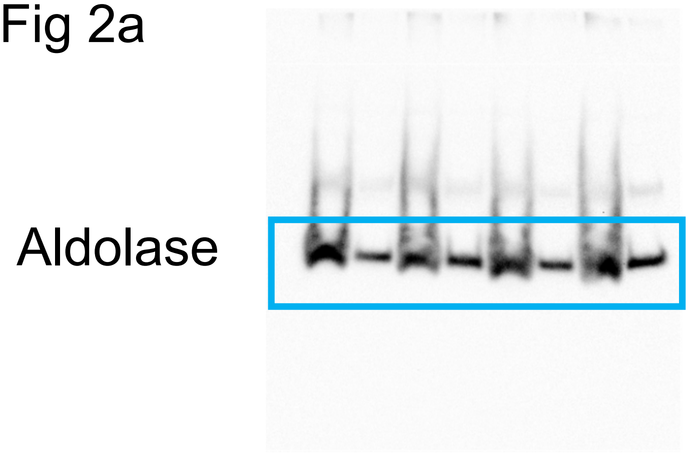

Fig 2c

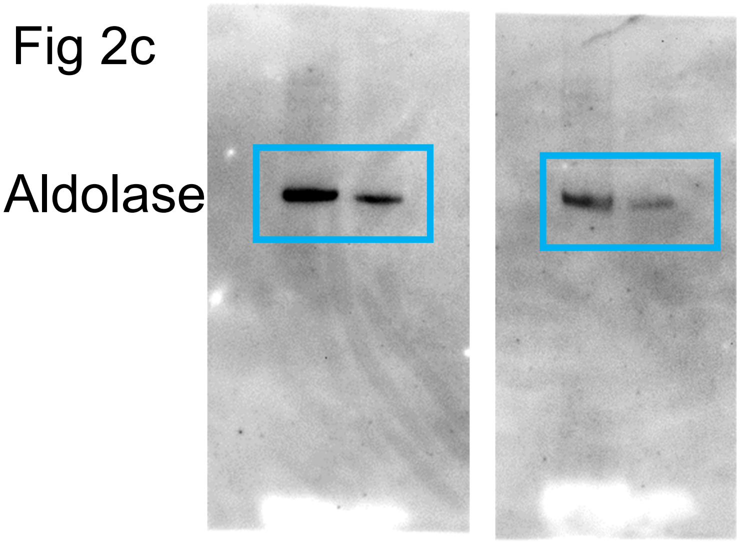

Fig 2d

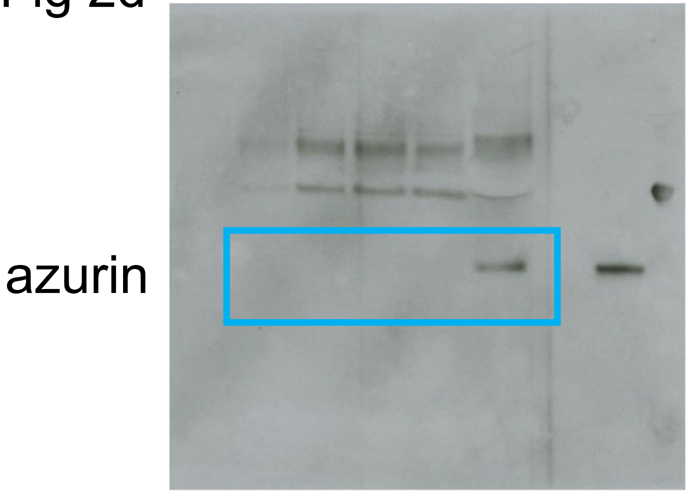

Fig 2e

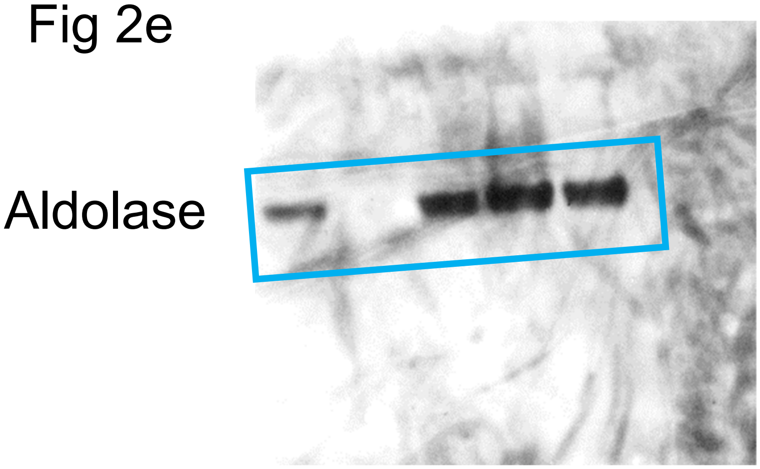

Fig 2f

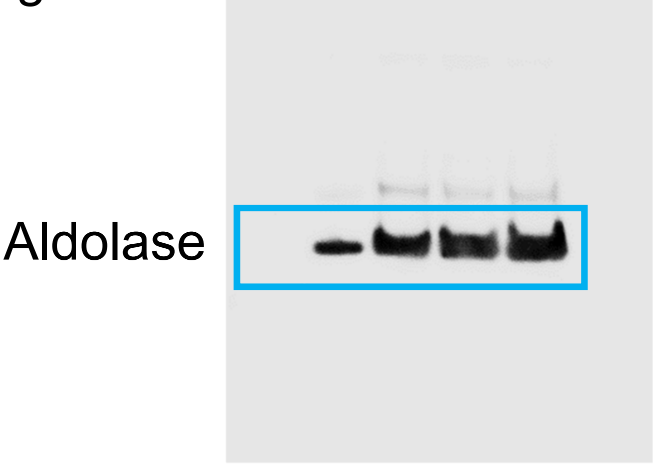

Fig 2g

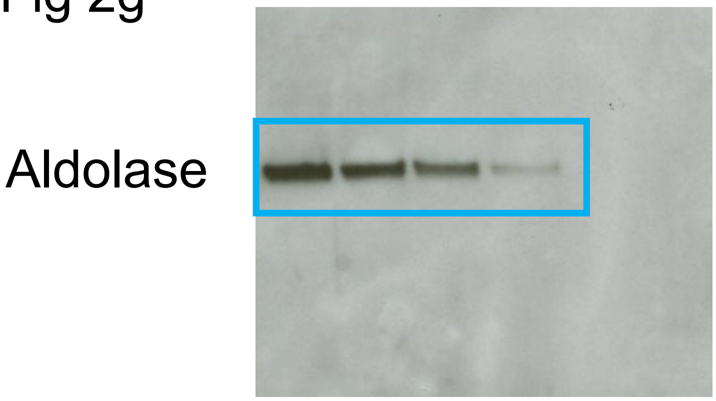

Fig 3d

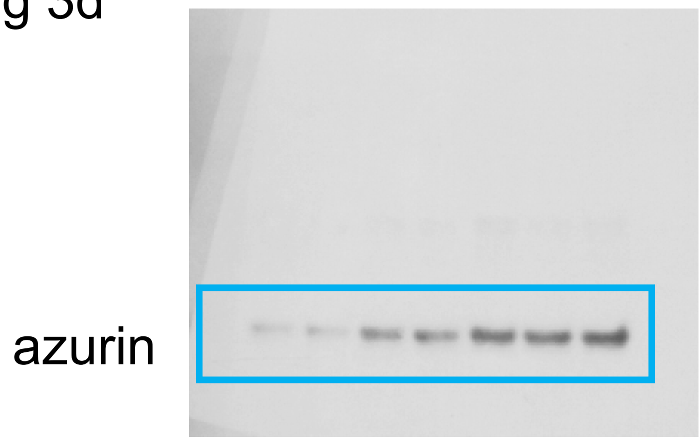

Fig 3e

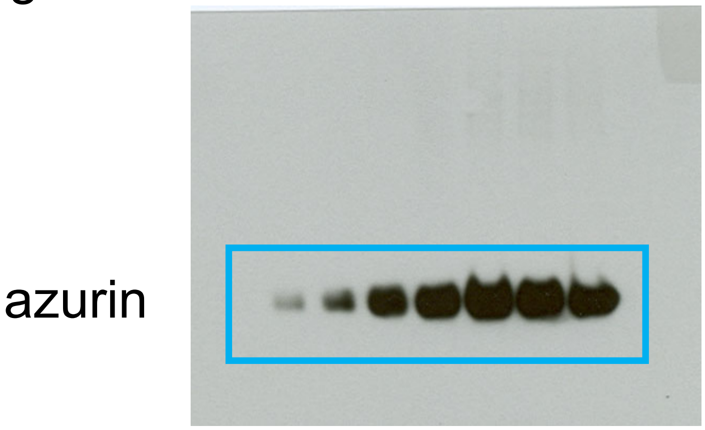

Suppl Fig 6

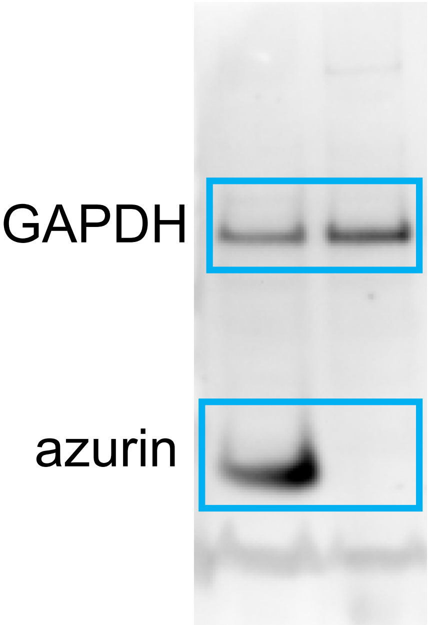

Suppl Fig 7

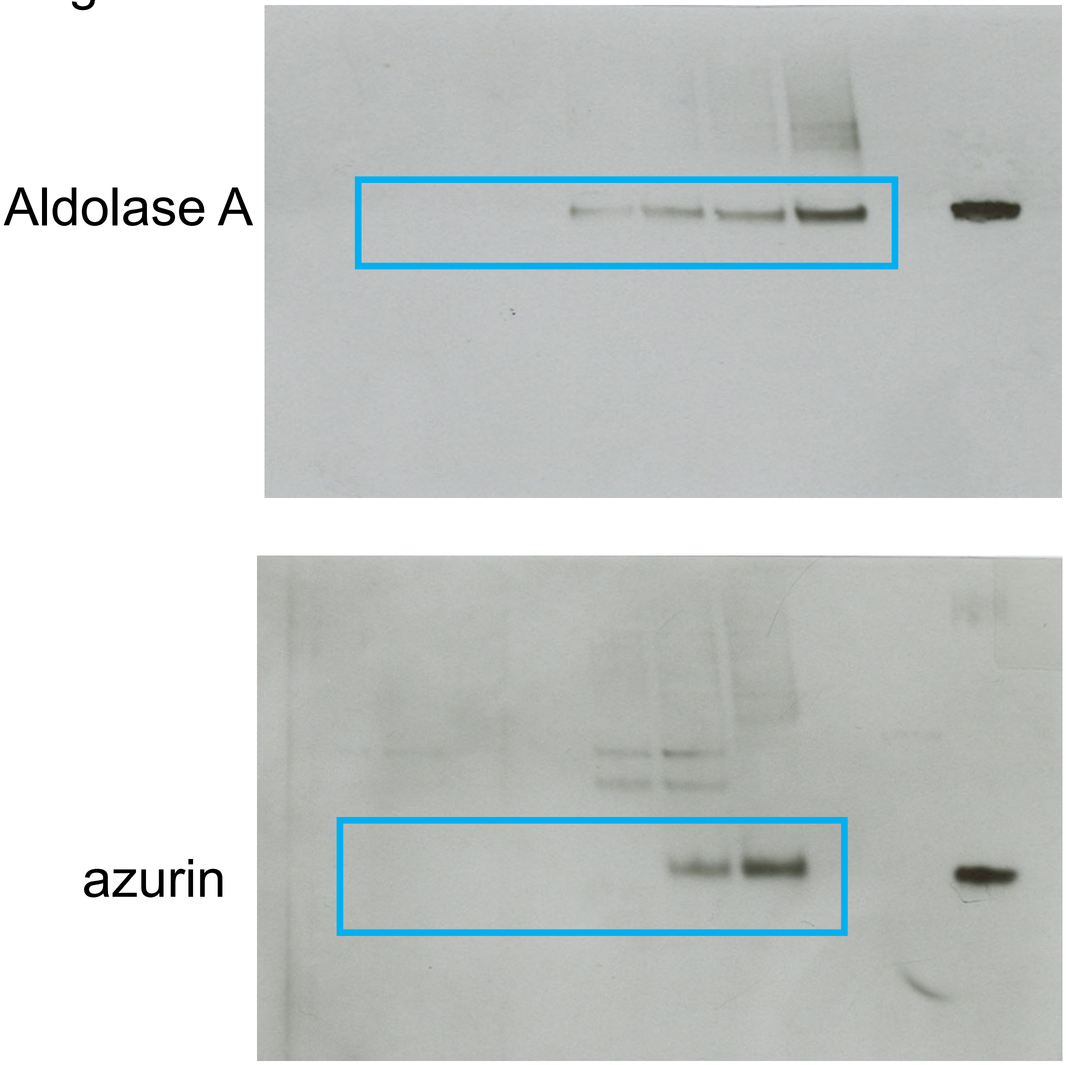

Suppl Fig 8

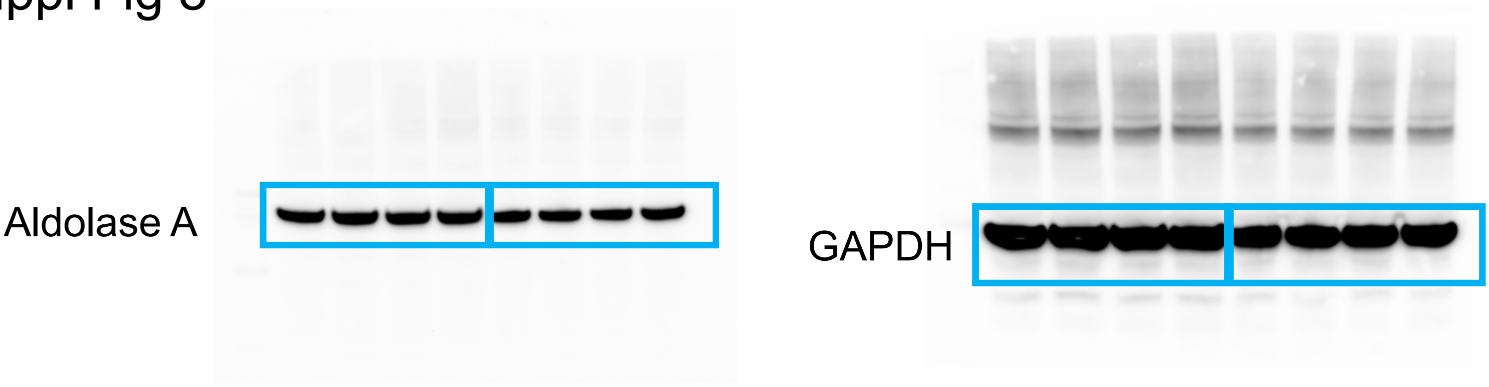

Suppl Fig 9

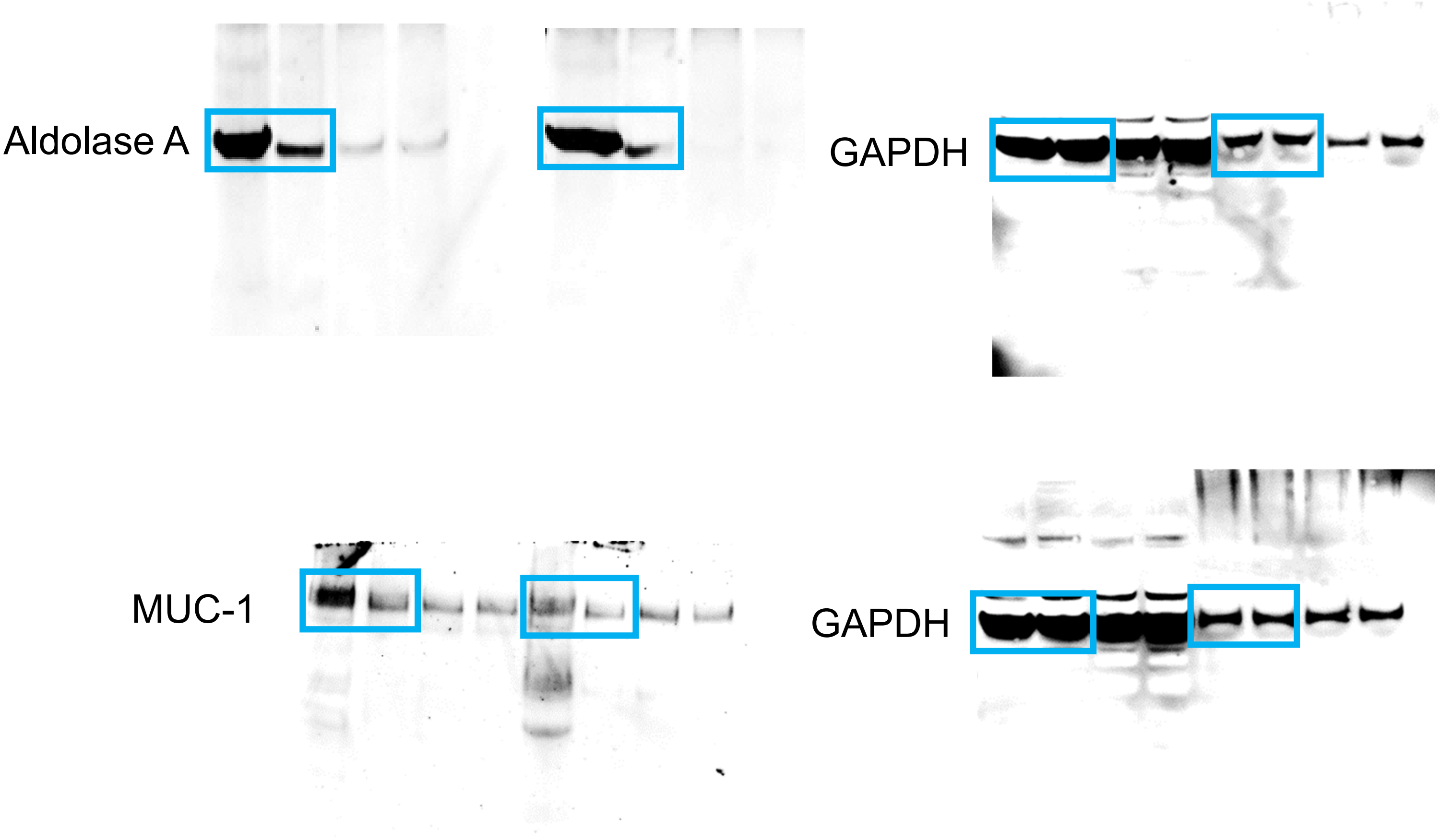

Suppl Fig 12

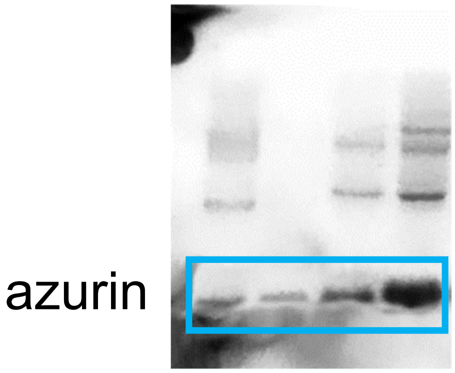

Supplement: Supplementary file 1 — Supplementary Information [file 42003_2022_4395_MOESM1_ESM.pdf]
