# Supplementary material for: Cross-talk between cancer and Pseudomonas aeruginosa mediates tumor suppression
Source: Commun Biol. 2023 Jan 6;6:16. doi: 10.1038/s42003-022-04395-5 (PMC9823004; doi:10.1038/s42003-022-04395-5)
Supplement: Supplementary file 2 — Reporting Summary [file 42003_2022_4395_MOESM2_ESM.pdf]

Corresponding author(s): Tapas K. Das Gupta and Tohru Yamada

Last updated by author(s): Nov 29, 2022

Reporting Summary

Nature Portfolio wishes to improve the reproducibility of the work that we publish. This form provides structure for consistency and transparency in reporting. For further information on Nature Portfolio policies, see our Editorial Policies and the Editorial Policy Checklist.

Statistics

For all statistical analyses, confirm that the following items are present in the figure legend, table legend, main text, or Methods section.

n/a	Confirmed	
☐	☒	The exact sample size (*n*) for each experimental group/condition, given as a discrete number and unit of measurement
☐	☒	A statement on whether measurements were taken from distinct samples or whether the same sample was measured repeatedly
☐	☒	The statistical test(s) used AND whether they are one- or two-sided<br>*Only common tests should be described solely by name; describe more complex techniques in the Methods section.*
☒	☐	A description of all covariates tested
☐	☒	A description of any assumptions or corrections, such as tests of normality and adjustment for multiple comparisons
☐	☒	A full description of the statistical parameters including central tendency (e.g. means) or other basic estimates (e.g. regression coefficient) AND variation (e.g. standard deviation) or associated estimates of uncertainty (e.g. confidence intervals)
☐	☒	For null hypothesis testing, the test statistic (e.g. *F*, *t*, *r*) with confidence intervals, effect sizes, degrees of freedom and *P* value noted<br>*Give P values as exact values whenever suitable.*
☒	☐	For Bayesian analysis, information on the choice of priors and Markov chain Monte Carlo settings
☒	☐	For hierarchical and complex designs, identification of the appropriate level for tests and full reporting of outcomes
☒	☐	Estimates of effect sizes (e.g. Cohen's *d*, Pearson's *r*), indicating how they were calculated

Our web collection on statistics for biologists contains articles on many of the points above.

Software and code

Policy information about availability of computer code

Data collection	Azurin detection in sera and tissues was performed by ELISA and PCR, respectively. Immuno blots were scanned and visualized. TEM images were taken by the Philips CM 120 transmission electron microscope. Breast tumor images were taken by the PDE neo imager.
Data analysis	Microsoft 365 Excel, band intensities determined by a densitometer UN-SCAN-IT gel version 5.1, GraphPad Prism ver. 8 and R (v.4.0.2) and RStudio (v.1.3.1093).

For manuscripts utilizing custom algorithms or software that are central to the research but not yet described in published literature, software must be made available to editors and reviewers. We strongly encourage code deposition in a community repository (e.g. GitHub). See the Nature Portfolio guidelines for submitting code & software for further information.

Data

Policy information about availability of data

All manuscripts must include a data availability statement. This statement should provide the following information, where applicable:

- Accession codes, unique identifiers, or web links for publicly available datasets
- A description of any restrictions on data availability
- For clinical datasets or third party data, please ensure that the statement adheres to our policy

The authors state that the data supporting the results of this study are available in this paper and supplementary materials. Extra data are available from the corresponding author upon request.

Field-specific reporting

Please select the one below that is the best fit for your research. If you are not sure, read the appropriate sections before making your selection.

☒ Life sciences ☐ Behavioural & social sciences ☐ Ecological, evolutionary & environmental sciences

For a reference copy of the document with all sections, see nature.com/documents/nr-reporting-summary-flat.pdf

Life sciences study design

All studies must disclose on these points even when the disclosure is negative.

Sample size	Power was calculated by using the R package pwr, and the sample size was estimated.
Data exclusions	No data were excluded from the analyses.
Replication	All attempts to repeat the experiments (>3 replicates) were successful.
Randomization	For animal studies, the mice were randomly divided into test groups.
Blinding	The researchers who conducted experiments (presented in Fig 4) were blinded to the sample details such as disease status, survival etc.

Reporting for specific materials, systems and methods

We require information from authors about some types of materials, experimental systems and methods used in many studies. Here, indicate whether each material, system or method listed is relevant to your study. If you are not sure if a list item applies to your research, read the appropriate section before selecting a response.

Materials & experimental systems

n/a	Involved in the study	
☐	☒	Antibodies
☐	☒	Eukaryotic cell lines
☒	☐	Palaeontology and archaeology
☐	☒	Animals and other organisms
☒	☐	Human research participants
☒	☐	Clinical data
☒	☐	Dual use research of concern

Methods

n/a	Involved in the study	
☒	☐	ChIP-seq
☒	☐	Flow cytometry
☒	☐	MRI-based neuroimaging

Antibodies

Antibodies used	Poly clonal anti-azurin Ab, monoclonal mouse anti-aldolase A (Santa Cruz Biotechnology, TX), mouse anti-Pseudomonas aeruginosa antibody (clone B11, Thermo Fisher Scientific, MA). Mouse Muc-1 (Santa Cruz Biotechnology).
Validation	Anti-azurin antibody was generated in our lab (Ref 1).<br>Purified azurin (Sigma-aldrich) was conjugated with keyhole limpet hemocyanin and generated a polyclonal antibody in rabbits (New Zealand White). Antibody titer was determined by direct ELISA using azurin (0-3 µg/well). An antibody dilution of 1:10,000 was sufficient to give a reproducible change in absorbance of 0.5 at 450 nm after 15 min incubation with substrate (1-Step PNPP; Pierce) at 25°C when 96-well plates (Nunc) were coated with azurin.<br>Other antibodies were validated by the vendor to specifically recognize the target molecule.

Eukaryotic cell lines

Policy information about cell lines

Cell line source(s)	MDA-MB-231, MCF10A, DU-145, CRL-11611, SK-OV3 and HOSE6-3 were obtained from the American Type Culture Collection). Human melanoma (UISO-Mel-2) and congenital melanocytic nevus (CMN) cell lines was developed in our laboratory as described.
Authentication	Cell authentication was provided by the vendor.
Mycoplasma contamination	All cell lines tested negative for mycoplasma.
Commonly misidentified lines (See ICLAC register)	No commonly misidentified lines were used in this study.

Animals and other organisms

Policy information about studies involving animals; ARRIVE guidelines recommended for reporting animal research

Laboratory animals	Hemizygous MMTV-PyMT transgenic mice were obtained from Jackson laboratories.
Wild animals	No wild animals were used in this study.
Field-collected samples	No field-collected samples were used in this study.
Ethics oversight	All experiments were approved by the Animal Ethics Committee of the University of Illinois at Chicago (UIC).

Note that full information on the approval of the study protocol must also be provided in the manuscript.
